# SPEI and SPI correlation in the study of drought phenomena in Umbria region (central Italy)

**DOI:** 10.1007/s11356-024-35740-2

**Published:** 2024-12-15

**Authors:** Sara Venturi, Daniel Dunea, Elena Mateescu, Ana Virsta, Nicolae Petrescu, Stefano Casadei

**Affiliations:** 1 Department of Civil and Environmental Engineering, University of Perugia, Via Duranti 93, 06125 Perugia, Italy; 2 Department of Environmental Engineering, Valahia University of Targoviste, Aleea Sinaia No.13, 130004 Targoviste, Romania; 3 National Administration of Meteorology, Șoseaua București-Ploiești 97, 013686 Bucharest, Romania; 4 Department of Environment and Land Reclamation, University of Agronomic Sciences and Veterinary Medicine of Bucharest, 59 Marasti Blvd., District 1, 011464 Bucharest, Romania

**Keywords:** Drought indices, Umbria region, SPEI-SPI correlation, Climate trend, ERA 5 reanalysis, CHIRPS, Climate Engine

## Abstract

This study aims to analyze the performances and correlation of the standardized precipitation index (SPI) and standardized precipitation evaporation index (SPEI) from the perspective of supplying effective indicators for drought risk management prevention. Indices have been evaluated using long time series of precipitation and temperature data (from 1961 to 2020) gauged and validated in the land monitoring system of the Umbria region (central Italy). Results show how SPEI can evaluate better the drought phenomena, both in terms of occurred events and in terms of trends. In particular, SPEI can appreciate the effects of the increase in temperatures, which in the next years could be predominant in c limate change. Currently, the high correlation between SPEI and SPI (*R*^2^ > 0.8 and *r* > 0.9) makes possible the use of SPI instead of SPEI in drought analysis; however, this correlation, evaluated on the two times series 1961–1990 and 1991–2020, shows a decreasing tendency; then, it could be no longer reliable in the future. These results should lead to increasingly synergistic monitoring of rainfall and temperature data, limiting as much as possible the lack of temporal overlap of the two sensors at the gauging stations. The possibility of using ERA 5 reanalysis data was also explored for the years that presented severe droughts by comparing them to the station-based observations. While the temperature data is reliable, the total precipitation parameter seems less affordable, and then, other available gridded datasets, e.g., CHIRPS, MERRA2, and Terraclimate, should have to be considered to improve the modeled precipitation’s suitability.

**Graphical abstract:**

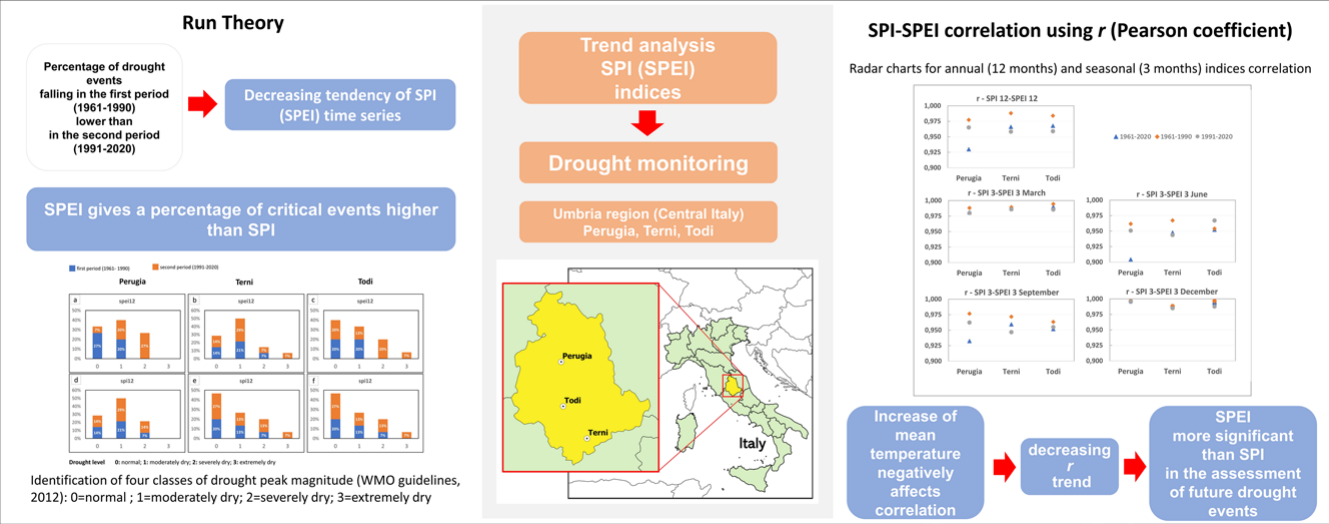

**Supplementary Information:**

The online version contains supplementary material available at 10.1007/s11356-024-35740-2.

## Introduction

The variability in hydroclimate has significantly intensified in recent years, with a higher frequency of extreme floods and drought events (Wang et al. 2022). One of the consequences of droughts is the significant influence on water resources management (Di Francesco et al. 2022, 2023; Citakoglu and Coşkun Ö (2022); Aktürk et al. 2024), with an impact on water quality and quantity (Dunea and Iordache 2011) and a potential massive backlash on water use (Peña-Guerreroa et al. 2020).

The focus of this study is to supply effective indicators from the perspective of drought monitoring management. The first step was the evaluation of long time series of data to detect the trend of SPI and SPEI in the last 60 years (from 1961 to 2020); the successive step was the estimation of the efficacy of using SPI instead of SPEI by testing the correlation between the two indices.

In literature, several studies on seasonality, trend, and variability of rainfall at different temporal and spatial scales (Mehta et al. 2022; Morbidelli et al. 2018, 2021: Citakoglu and Minarecioglu 2021; Caloiero et al. 2018) are available, since the lack of precipitation is considered the main reason of drought. Some of these studies investigated the precipitation employing the standardized precipitation index (SPI) (McKee et al. 1993). In this regard, SPI was recommended as the worldwide standard index by the World Meteorological Organization and the Lincoln Declaration on Drought (Hayes et al. 2011) and was introduced by the Water Scarcity and Drought Expert Group of the European Commission within the set of common indicators for water scarcity and drought (Faergemann 2012).

In recent years, SPI has been employed in various studies to determine drought conditions across the world at different spatial and temporal scales. Romano et al. (2022) studied the meteorological drought events in central Italy, and the increase of the oscillations in the precipitation regime has been highlighted by a 24-month Standardized Precipitation Index (SPI 24). Citakoglu and Coşkun (Citakoglu and Minarecioglu 2021; Coşkun and Citakoglu 2023) implemented models to predict the future values of the SPI drought indices for 1-, 3-, and 6-month time scales of Sakarya province located in the northwest of Turkey. Kebaili and Jemai (2022) focused on SPI with a 3-month accumulation (SPI 3) for quantifying the immediate drought impacts, such as agricultural and soil moisture drought in a Tunisian basin, Medjerda River Basin.

The 3-month aggregation of the index has usually been used to define seasonal series (Crespi et al. 2020). Specifically, in the Northern Hemisphere, the seasonal time series were defined by considering SPI 3 values in February for winter, May for spring, August for summer, and November for autumn.

The relevance of SPI in drought studies is proved by the existence of several monitoring systems based on the SPI survey such as the National and Oceanic Atmospheric Administration (NOAA). However, under global climate warming, air temperature variations are expected to become the new primary driver of droughts, in particular in high-latitude cold catchments. Gu et al. (2022) highlighted an increased risk of global extreme hydrological droughts due to warming and suggested that rising temperatures at high latitudes may lead to more extreme hydrological droughts. It is also evident that climate change-induced warming has accelerated the hydrological processes, firstly, by increasing temperatures and energy available for evapotranspiration (ET). This explains ET’s important role in releasing droughts and drought severity at both local and global scales Hargreaves and Allen (2003). Therefore, using ET together with precipitation P in the structure of drought indices allows for a more comprehensive drought assessment (Khoshnazar et al. 2022).

The standardized precipitation evapotranspiration index (SPEI) was first proposed by Vicente-Serrano et al. (2010) and uses the basis of SPI but overcomes the limitation of using solely precipitation, as it is based on ET calculation. Input parameters are monthly precipitation and temperature data, and a complete time series of data is required with no missing months.

SPEI has been employed in different areas of the world to analyze drought incidence and its relationships with water scarcity. For example, Ndayiragije and Li (2022) have studied SPEI in Burundi to determine drought occurrences and their characteristics in terms of duration, severity, intensity, and analysis of trends; Jincy Rose and Chithra (2022) have extended the study of SPEI to investigate the statistical relation between SPEI and hydrological drought indices (Standardised Streamflow Index and Streamflow Drought Index) in the Bharathapuzha river basin of Kerala, India; Vergni et al. (2021) have tested the efficacy of some standardized meteorological indices in the identification of agricultural drought, observing that SPEI presents the highest correlation with a new index, the Standardized Deficit Distance Index (SDDI); Fan et al. (2018) have used copulas to quantify the relationship between drought and water scarcity based on different indicators (SPEI and Water Exploitation Index plus (WEI^+^)), based on their joint probability distribution.

Even if the utility of SPEI in drought studies is demonstrated (Li et al. 2020), the study of correlations between SPEI and SPI is crucial to overcome the requirement of a complete dataset for both temperature and precipitation. Therefore, the analysis of the SPEI vs. SPI correlation of stations in Umbria, central Italy, becomes a strong point of this study.

At present, only a small number of studies dealing with this topic are present in the literature (Sharafati et al. 2020; Lotfirad et al. 2021; Tirivarombo et al. 2018) and sometimes do not agree with each other. The first two studies focused on Iran’s regions characterized by various climate conditions to evaluate if the correlation is climate-dependent. Lotfirad et al. showed that the correlation between the SPI and SPEI is lower in arid climatic areas, in agreement with the results of Tirivarombo et al. Instead, Sharafati et al. assessed a high correlation between the SPI and SPEI in all climatic regions.

We underline that we have chosen to use data from the ground-based weather stations, and not rely only on the gridded datasets, since the previous studies refer that such datasets can produce a deviation in the estimation of trend magnitude of hydrologic parameters and uncertain results for areas with complex topography (My et al. 2022; Sidau et al. 2021). Blankenau et al. (2020) assessed the quality of the gridded data set (i.e., RTMA; NLDAS) for modeling the reference evapotranspiration, concluding that it is generally overestimated including positive biases of several air parameters, in particular in the temperature.

Anyway, as further analysis, we have tested the possibility of using data extracted from the ERA 5 reanalysis dataset, i.e., temperature and total precipitation, for the years that presented severe droughts in the last 60 years (from 1961 to 2020) in the Umbria Region. ERA 5 datasets were compared to the station-based observations at a daily scale using the Willmott Index of agreement (Willmott 1981) to explore the scalability of the application. Furthermore, we have analyzed the SPEI maps obtained from MERRA2, ERA5, and Terraclimate datasets using the application from the Climate Engine (https://app.climateengine.org/climateEngine#).

## Materials and methods

### Drought monitoring based on standardized indices

One of the most used indices for monitoring droughts, first developed by Mckee et al. (1993), is the Standard Precipitation Index (SPI). The wide and commonly accepted application is due to the relatively straightforward computation since SPI requires only a single data series of long-term precipitation as input (Svoboda et al. 2012).

In 2010, Vicente-Serrano et al. proposed a new index, the Standardized Precipitation Evapotranspiration Index (SPEI). SPEI is calculated similarly to SPI but integrates the impact of temperature changes as part of the analysis. It preserves the simplicity, multiscale nature, and statistical interpretability of the SPI but includes the influence of Potential Evapotranspiration (PET) (Dunea et al. 2021).

SPI/SPEI time series can be used for drought events assessment after setting a specific threshold, to define the start and ending points of droughts. A common choice is setting the threshold to zero, according to the document: “Overview of Drought Monitoring Indicators and Indices” (World Meteorological Organization 2024), which defines the drought event as ongoing until SPI (or SPEI) from negative values reaches a value of zero.

For the duration of the event, summarized values of SPI and SPEI can be used to define drought severity.

For both indices, it is generally considered that the computation needs at least 30 years of data to be considered reliable; however, longer samples can assure better accuracy in drought estimations (Wu et al. 2005).

In agreement with Mackee et al. (1993) and Svoboda et al. (2012), seven different SPI classes can be identified (Table S1 in Supplementary). In literature, it is generally accepted to adopt identical classes for SPEI (Herold et al. 2018; Tefera et al. 2019). As SPI/SPEI is a standardized variable, it is possible to associate the theoretical occurrence probability with each class (Kamruzzaman et al. 2022).

In this study, both SPI and SPEI values for different time scales were calculated in the R-environment using the SPEI package https://cran.r-project.org/package=SPEI. In this package, the function allowing to compute SPI is identical to the SPEI function, except for the distribution. The computation methodology for both indices is described in Supplementary (sections S.1.1 and S.1.2).

We have described seasonal and annual time series with 3- and 12-month indices (hereafter named SPI 3/SPEI 3 and SPI 12/SPEI 12, respectively), following the approach of Crespi et al. (2020). Specifically, SPI 3 and SPEI 3 in March characterize the winter season, June the spring, September the summer, and December the autumn. For the annual series, the SPI 12 and SPEI 12 values in December for each year have been considered.

### Drought features identification—runs theory

**Fig. 1:**
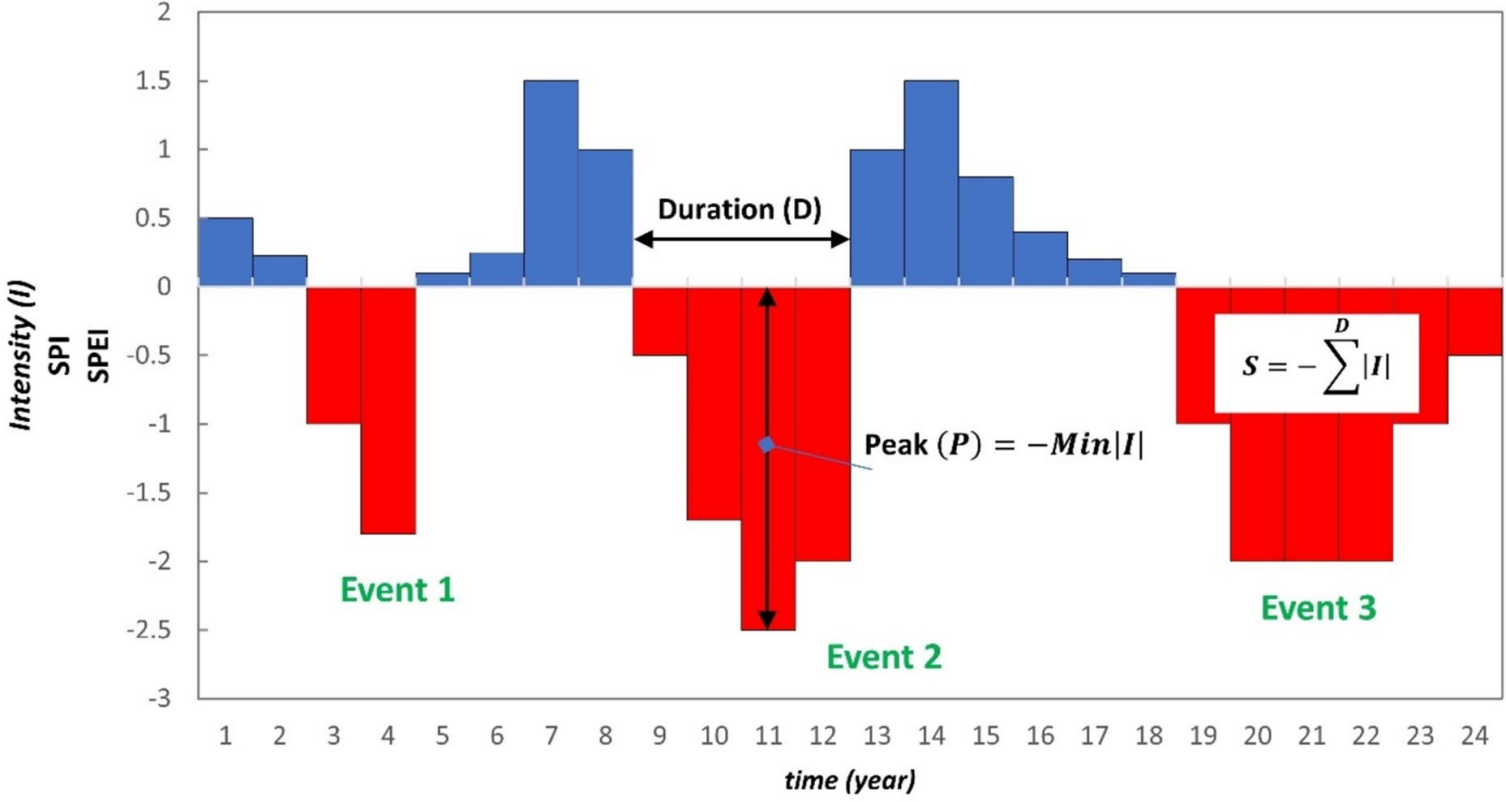
Drought characteristics extraction using runs theory for different events

In this study, we have chosen to identify drought events using the runs theory as described in Yevjevich (1967). SPI and SPEI values are characterized as time series functions and specify the intensity (*I*) of drought (or wet) conditions. Continuous negative values of the index under a threshold value indicate that a drought event occurs. In Figure 1, the method to define the characteristics of each drought event (severity, peak, and duration) is shown. Duration (*D*) is the number of periods of uninterrupted negative SPI or SPEI, the peak (*P*) is the highest intensity of a certain drought event, and *S* (severity) is the sum of drought intensity for a certain drought event. Previous concepts of *P* and *S* can be expressed using formulas:

1 $$P=-\text{Min} \left|{I}_{\text{SPI},\text{SPEI}}\right|$$

2 $$S=-{\sum }_{i=1}^{D}{\left|{I}_{\text{SPI},\text{SPEI}}\right|}_{i}$$

where *D* is expressed in years and must be considered the number of consecutive years in which the value of the specific drought event has remained under a certain threshold.

The run theory has been applied in the study of SPI and SPEI time series on the base of free software under a GPL-3.0 license (https://github.com/adrHuerta/drought_features).

### Methods for trend detection

In this study, the significance of the indices trend has been evaluated following both Mann-Kendall and Innovative-Sen trend methods and results have been compared.

The Mann-Kendall (MK) method (Mann 1945; Kendall 1975) is a non-parametric test to analyze the trend in time series, under the hypothesis that data are independent and identically distributed (Hamed 2008). More details can be found in Supplementary S.1.3.

The Innovative-Sen trend (IST) method for trend detection was first proposed by Sen (2012). The test is applied by splitting time-series data into two subsets and then arranged in ascending order in a cartesian coordinate system. The 1st half series is plotted on the *x*-axis (horizontal axis), and 2nd half series is plotted on the *y*-axis (vertical axis). An upward or a downward trend exists in the time series if data fall, respectively, above or below the 1:1 line. If the scatter points of the data appear on the 45° straight line or close to this line, it means that there is no significant trend (no-trend time series) (Sen 2012, 2017; Dabanlı et al. 2016). Details regarding the trend assessment using the IST can be found in section S.1.4 in Supplementary.

Malik et al. (2020) showed that, for a certain data set, the IST test can identify the significance of a monotonic trend in several cases higher than the MK method and that the identified trend is more reliable. Similar conclusions were reported by Harka et al.(2021), who applied the IST method in the hydro-meteorological analysis of time series, and by Zhou et al., (2018), who studied the trends of annual and seasonal solar radiation in several stations in China between 1962 and 2015.

## Case study

### Study area

In this study, we have used data collected from weather stations with measurements of precipitation and air temperature characterized by long time series (from 1961 to 2020).

**Fig. 2:**
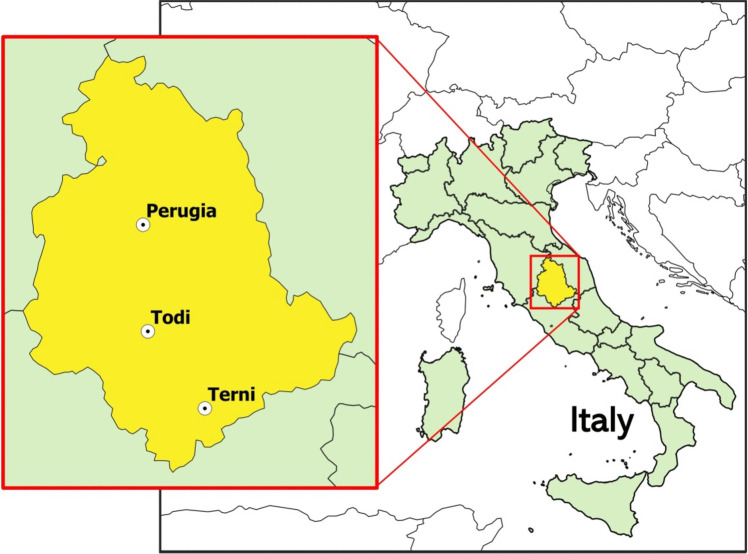
The geographic position of the stations: Perugia, Terni, and Todi (Italy)

The weather stations of Perugia, Terni, and Todi are in the Umbria region, central Italy (Figure 2).

Umbria region covers 8456 km^2^, its area is mainly hilly and partially dominated by the Apennine reliefs, with more than 40% of the territory covered by forests. Urban areas, roads, and highways are principally placed along the Tiber River Valley that extends from North to South of the region and the wide valley between the towns of Perugia and Spoleto (Figure S3 in Supplementary). The Tiber basin is characterized by an area of around 17,375 km^2^ and travels about 409 km before draining into the Tyrrhenian Sea (Cesari 2010).

The region is characterized by a mean annual rainfall of about 900 mm, differing from 650 to 1450 mm depending on the geographic position. Generally, the mean monthly temperature fluctuates between 25.5 and 6 °C, 24.41 and 5.54 °C, and 23.33 and 5.37 °C, respectively, in Perugia, Terni, and Todi. Generally, the maximum value is reached in July or August, and the minimum in January, respectively.

### Data source and data processing

Data from Umbria weather stations of Perugia, Terni, and Todi are acquired from the Regional Hydrographic Service (http://www.annali.regione.umbria.it/) and derived from the automatic monitoring sensors network.

In Table S2 (Supplementary), the main characteristics of the rainfall-temperature weather stations are presented: geographic position, type of available data, and availability periods.

Data from Perugia station have been derived by integrating the ones of the old station at S.Pietro Church with the ones at Perugia Fontivegge and Santa Giuliana, which are all close to each other and characterized by a similar altitude. The data collected from the three stations can be considered acceptable in the context of the present study, in which rainfall and temperature values are aggregated at 3- and 12-month time scales to derive drought indicators.

We specify that the monthly rainfall data used for both SPI and SPEI calculation has been obtained as cumulative daily rainfall; the mean, maximum, and minimum monthly temperatures are an average of mean, maximum, and minimum daily temperatures.

### ERA 5 reanalysis dataset

We used the Climate Data Store capabilities to extract data at given locations from the ERA5 dataset (https://cds.climate.copernicus.eu/cdsapp#!/dataset/reanalysis-era5-single-levels?tab=overview). The information is provided and updated by the Copernicus Climate Change Service (C3S), which is implemented by the European Centre for Medium-Range Weather Forecasts (ECMWF) on behalf of the European Commission (Lavers et al. 2022). ERA5 is the fifth generation ECMWF reanalysis for the global climate and weather for the past 8 decades. Data is available from 1940 onwards. More details related to the ERA 5 datasets including the models and interpolation methods can be found at https://confluence.ecmwf.int/display/CKB/The+family+of+ERA5+datasets.

A script was developed to extract the selected variables (“2m_temperature” and “total_precipitation”) through the CDS API for the years 1975, 1983, 2001, 2003, 2007, 2015, and 2017 (showing the severe droughts in the Umbria region) and for the corresponding geographic coordinates of the considered meteorological stations based on the interpolation capabilities of the Meteorological Interpolation and Regridding model (MIR software). Data were processed to adjust the time scale (daily averages) and the units of measurement (Kelvin degrees to Celsius degrees for temperature and m to mm for precipitations amount). The extracted time series were compared with the daily time series from the meteorological stations using the Willmott Index of agreement (1981), *d*. These indices are sensitive to variations between measured and modeled values and can assess the degree to which the model can capture the measured variance.

The Climate Engine application was used to obtain SPEI thematic maps based on three datasets (https://app.climateengine.org/climateEngine#) for three selected years for comparison i.e., 2000, 2010, and 2020.

## Results

The following sections “Precipitation and temperature: evaluation and comparison of their trend,” “SPEI and SPI trends,” and “Run test”, dealing with the trends analysis of rainfall, temperature, and indices are important both to understand the results of the run test (“ERA 5 reanalysis dataset” section) and to underline the relevance of the study of correlations between SPEI and SPI (“SPEI and SPI correlation” section).

### Precipitation and temperature: evaluation and comparison of their trend

In this study, the climatic characteristics of Perugia, Terni, and Todi are compared, taking into consideration the annual cumulative precipitation (ACP) and the annual average temperature (AAT).

As it is possible to observe in Figure S4 (Supplementary), the ACP range for Umbria stations is between 430.4 mm (minimum value for Terni) and 1283.2 mm (maximum value for Perugia). The AAT of Terni is higher for both minimum and maximum values compared to the remaining Umbria stations.

For each station, Table 1 includes the slope of linear regression ($m$), standardized variable *Z*_MK_, slope *s*, and the correspondent confidence limit determined using the IST test.

**Table 1:** Linear regression, MK test, and IST method applied to annual cumulative precipitation and average annual temperature at the station: Perugia, Terni, and Todi

Station	Variable	Linear regression (slope *m*)	Mann Kendal test (standardized variable, *Z*_MK_)	IST—trend slope	
				Confidence limit (${CL}_{1-\alpha }$)	Trend slope (*s*)
Perugia	Annual cumulative precipitation	0.109	− 0.044	± 0.237	1.377*
Terni		− 2.28	− 1.85	± 0.412	− 2.086*
Todi		− 2.13	− 1.47	± 0.241	− 2.219*
Perugia	Average annual temperature	0.0433	6.9*	± 0.003	0.045*
Terni		0.0179	3.93*	± 0.002	0.009*
Todi		0.0327	5.96*	± 0.001	0.035*

*Statistically significant at a 5% level

First, considering linear regression slope, Terni and Todi present an evident decreasing tendency of ACP ($m$ equal to − 2.28, and − 2.13, respectively, for Terni and Todi). Differently, Perugia shows a slightly increasing tendency of ACP ($m$ = 0.109). In Table 1, it is also possible to observe that all the stations present an increasing trend of AAT, ($m$ equal to 0.0433, 0.0179, and 0.0327, respectively, for Perugia, Terni, and Todi).

Then, the MK test and the IST method allowed us to assess the significance of the monotonic trend of ACP and AAT. MK test evaluates the trend of the ACP of Terni and Todi not significant, for a significance level of 5%. However, it has been proved that the MK test needs too restrictive assumptions in detecting trends of hydrological variables, and, in several conditions, the IST is preferable. The decreasing trend of AAT is evaluated both by the MK test and IST method as significant, for a level of 5% (Table 1).

It is possible to conclude that Terni and Todi present a monotonic trend, decreasing for precipitation and increasing for temperature. Perugia differs for a slightly increasing tendency of precipitation, though it is barely appreciable, and the slope $s$ of the IST method is very low and less far from the confidence limit concerning other stations’ slopes.

### SPEI and SPI trends

The tendency of annual and seasonal time series is shown in Figure S5 (supplementary): for each station, in the same chart are represented both SPI and SPEI temporal series (respectively, the red and blue line). For SPI 12 and SPEI 12 and seasonal SPI 3 and SPEI 3, Figure S6 and Figure S7 illustrate the linear regression analysis with the correspondent regression equation; Figure S8 and Figure S9 the results of the IST method, for all the stations. Tables 2 and 3 allow the comparison of the results of regression analysis, MK, and IST methods applied to SPI and SPEI indices.

**Table 2:** SPI, SPEI 12 (linear regression, MK test, IST)

Station	Indicator	Linear regression (slope *m*)	MK test (standardized variable *Z*_MK_)	IST—trend slope	
				Confidence limit (${CL}_{1-\alpha }$)	Trend slope (*s*)
Perugia	SPI 12	− 0.0002	− 0.04	± 0.00122	0.00681*
Terni		− 0.0127	− 1.86	± 0.00198	−0.01211*
Todi		− 0.0121	− 1.47	± 0.00153	−0.01280*
Perugia	SPEI 12	− 0.0173	− 2.52*	± 0.00169	−0.01232*
Terni		− 0.0104	− 1.55	± 0.00165	−0.00612*
Todi		− 0.0175	− 2.25*	± 0.00140	−0.01933*

*Statistically significative at a 5% level

**Table 3:** SPI, SPEI 3—March, June, September, December (linear regression, MK test, IST)

Station	Indicator	Linear regression (slope *m*)	MK test (*Z*_MK_)	IST—trend slope	
				Confidence limit (${CL}_{1-\alpha }$)	Trend slope (*s*)
Perugia	SPI 3 (mar)	0.0008	0.172	± 0.00101	−0.00169*
Terni		− 0.0076	− 0.887	± 0.00140	− 0.01391*
Todi		− 0.0067	− 0.453	± 0.00209	− 0.01600*
Perugia	SPEI 3 (mar)	− 0.0029	− 0.364	± 0.00152	− 0.00658*
Terni		− 0.0085	− 0.950	± 0.00178	− 0.01448*
Todi		− 0.0071	− 0.542	± 0.00152	− 0.01623*
Perugia	SPI 3 (jun)	0.0085	1.384	± 0.00117	0.00953*
Terni		− 0.0075	− 0.950	± 0.00262	− 0.00648*
Todi		0.0018	0.198	± 0.00125	− 0.00036
Perugia	SPEI 3 (jun)	− 0.0104	− 1.346	± 0.00173	− 0.01119*
Terni		− 0.0028	− 0.338	± 0.00261	0.00102
Todi		− 0.0053	− 0.504	± 0.00195	− 0.00879*
Perugia	SPI 3 (sept)	− 0.0093	− 1.601	± 0.00268	− 0.00060
Terni		− 0.0021	− 0.338	± 0.00247	0.00138
Todi		− 0.0081	− 0.721	± 0.00187	− 0.00775*
Perugia	SPEI 3 (sept)	− 0.0235	− 3.374*	± 0.00263	− 0.01806*
Terni		0.0006	− 0.338	± 0.00230	0.00538*
Todi		− 0.0151	− 1.588	± 0.00244	− 0.01664*
Perugia	SPI 3 (dec)	0.0009	0.14	± 0.00213	0.00775*
Terni		− 0.0038	− 0.66	± 0.00189	− 0.00102
Todi		− 0.0084	− 0.64	0.00343	− 0.00307
Perugia	SPEI 3 (dec)	− 0.0019	− 0.15	± 0.00229	0.00521*
Terni		− 0.0050	− 0.66	± 0.00170	− 0.00068
Todi		− 0.0073	− 0.62	± 0.00262	− 0.00161

*Statistically significant at a 5% level

First, both for SPI and SPEI 12, it is possible to observe that the slopes of linear regression $m$ are negative for all the stations, indicating a general negative trend. Differences in regression slopes between the stations can be evaluated in Table 2: SPI 12 presents comparable values for Terni and Todi (respectively, − 0.0127, − 0.0121), and a very low value (− 0.0002) for Perugia, consistently with the precipitation trend of Table 1. Differently, considering SPEI 12, Perugia presents a slope value comparable with Todi (respectively, the regression slope is − 0.0173, − 0.0175), instead, Terni has a lower value (− 0.0104), probably due to a higher temperature over the entire period (Figure S4).

Table 2 outlines the values of the standardized variable (${Z}_{\text{MK}}$) of MK test for SPI 12 and SPEI 12. MK test provides a significant negative monotonic trend only for the SPEI 12, not for the SPI 12, although the value of ${Z}_{\text{MK}}$ for Terni (− 1.87) is close to the threshold (± 1.96). Differently, the IST method provides a significant negative monotonic trend both for SPI 12 and SPEI 12.

Then, considering the seasonal SPI 3 in Figure S10, Perugia generally shows a low positive regression slope: 0.0008 in March (b), 0.0085 in June (c), and 0.0009 in December (d) except in September (−0.0093).

Table 3 outlines the values of the standardized variable (${Z}_{\text{MK}}$) of MK test and *s* trend slope from IST method, for seasonal SPI 3 and SPEI 3. Similarly to the SPI 12 and SPEI 12 trends, the MK test hardly considers the monotonic trend of the SPI 3 and SPEI 3 time series significant. An exception occurs only in September for SPEI 3 of Perugia. Differently, the IST method provides generally a significant negative monotonic excepting the following: in June, SPI 3 in Todi and SPEI 3 in Terni; in September, SPI 3 in Perugia and Terni; in December, SPI 3 and SPEI 3 in Terni and Todi.

### Run test

The run test has been used to identify the drought features based on SPI/SPEI time series (3 and 12 months), considering the first half part of the time series (first period: FP, 1961–1990) and the second half part (second period: SP, 1991–2020).

A zero threshold for both SPI and SPEI indices has been chosen for run test analysis to detect drought events and extract the characterizing parameters: severity, duration, and peak. This choice is in agreement with the studies of Sharafati et al. (2019) and Lotfirad et al. (2021) who used the run test analysis to reconstruct the meteorological drought features. It is pointed out that the duration is the number of years in which the value of a specific drought event has continuously remained under a certain threshold.

**Fig. 3:**
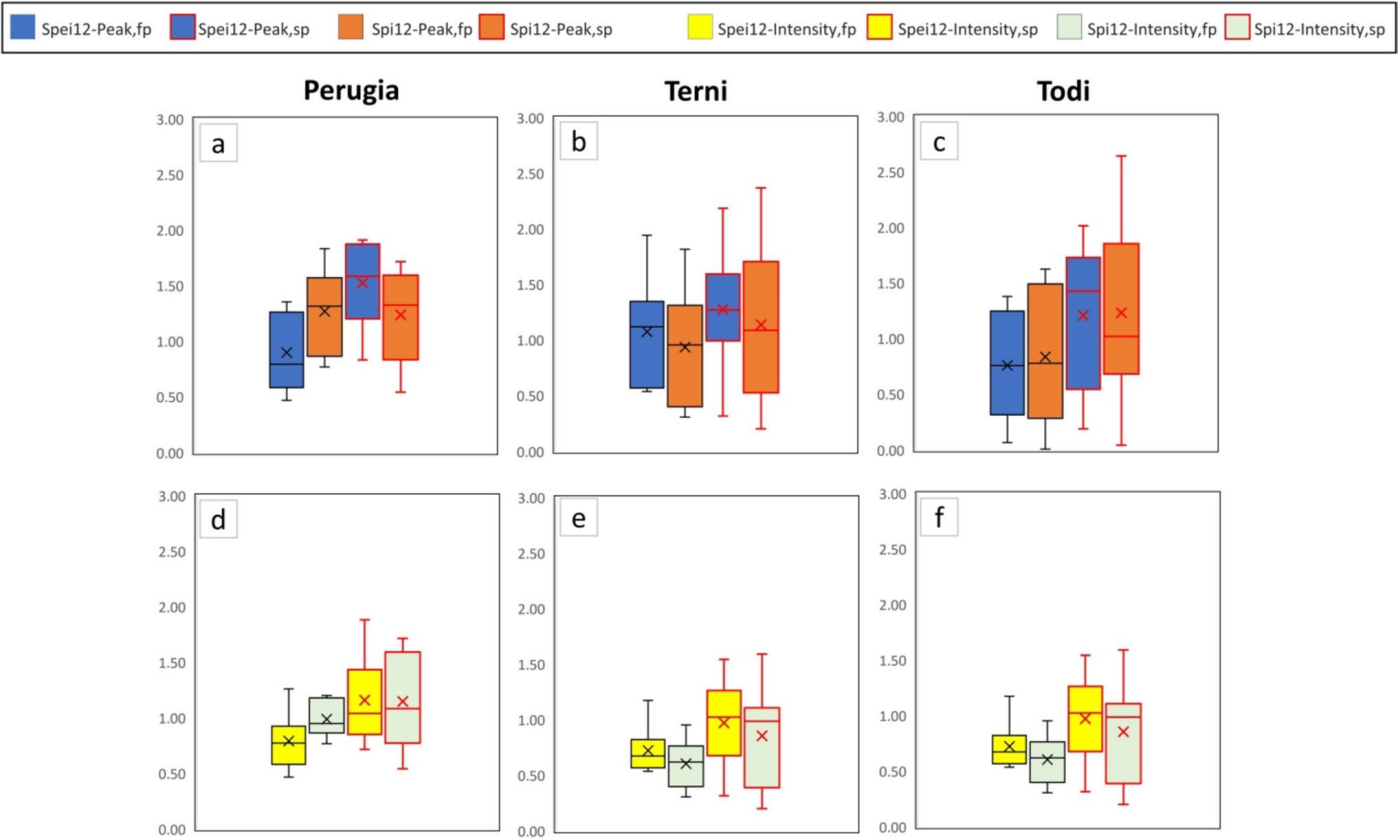
Boxplot of the absolute value of peak and intensity for both SPEI and SPI 12 for each station. Boxplot elements: box = values of 25th and 75th percentiles; horizontal line = median; cross = average; whiskers limit = minimum/maximum value; interquartile range (IQR) = difference between 25 and 75th percentile

To completely describe the drought events in the two periods (FP, SP), for an accumulation time of 3 months and 12 months, in Table S3, S4, S5, S6, and S7 (supplementary), the following parameters have been listed: number of events, maximum, average and standard deviation (SD) of the duration, maximum peak, and severity value and their occurrence. Then, in Figure 3 and S11 (supplementary), the boxplot charts of the peak and intensity magnitudes of each drought event are shown.

First of all, the median (average) of both peak and intensity is lower in the first period than in the second period. An exception to the previous consideration can be found in Figure 3a: in fact, SPI12 of Perugia has a similar median (average) of peak and intensity value in the first and second periods, due to the not significant trend that characterizes the rain along the whole observation period. Then, it is possible to observe that, in the majority of cases, in the first period, the difference between the intensity and peak median (average) derived from SPEI and SPI varies randomly; differently, in the second period, the median (average) from SPEI generally overcomes the SPI one. An exception to the previous consideration is the intensity of Perugia shown in Figure 3d.

For a 3-month accumulation period (Figure S11, supplementary), the previous observations are generally effective, even if a few exceptions jump out. For instance, considering the SPI 3 in September, Perugia presents a median magnitude of the peak of the SPI 3 lower in the second than in the first period (Figure S11, m). Another exception can be found in the peak median (average) of SPEI 3 that, in the second period, does not overcome the SPI 3 one in March (Figure S11 supplementary, b) and December (Figure S11 supplementary, t and u).

From the aforementioned observations, it would be possible to conclude that drought features such as peak and intensity, in the second period, show a magnitude higher than in the first period, following the negative trend of SPI and SPEI. Moreover, in the second period, usually, the run test analysis of SPEI provides higher values of peak and intensity than SPI. This tendency in the first period is not clearly defined. The previous consideration is in agreement with the trend test analysis of SPI/SPEI and can be explained by the fact that SPEI, which takes into consideration the temperature, can capture, better than SPI, the increasing trend of the drought phenomena.

**Fig. 4:**
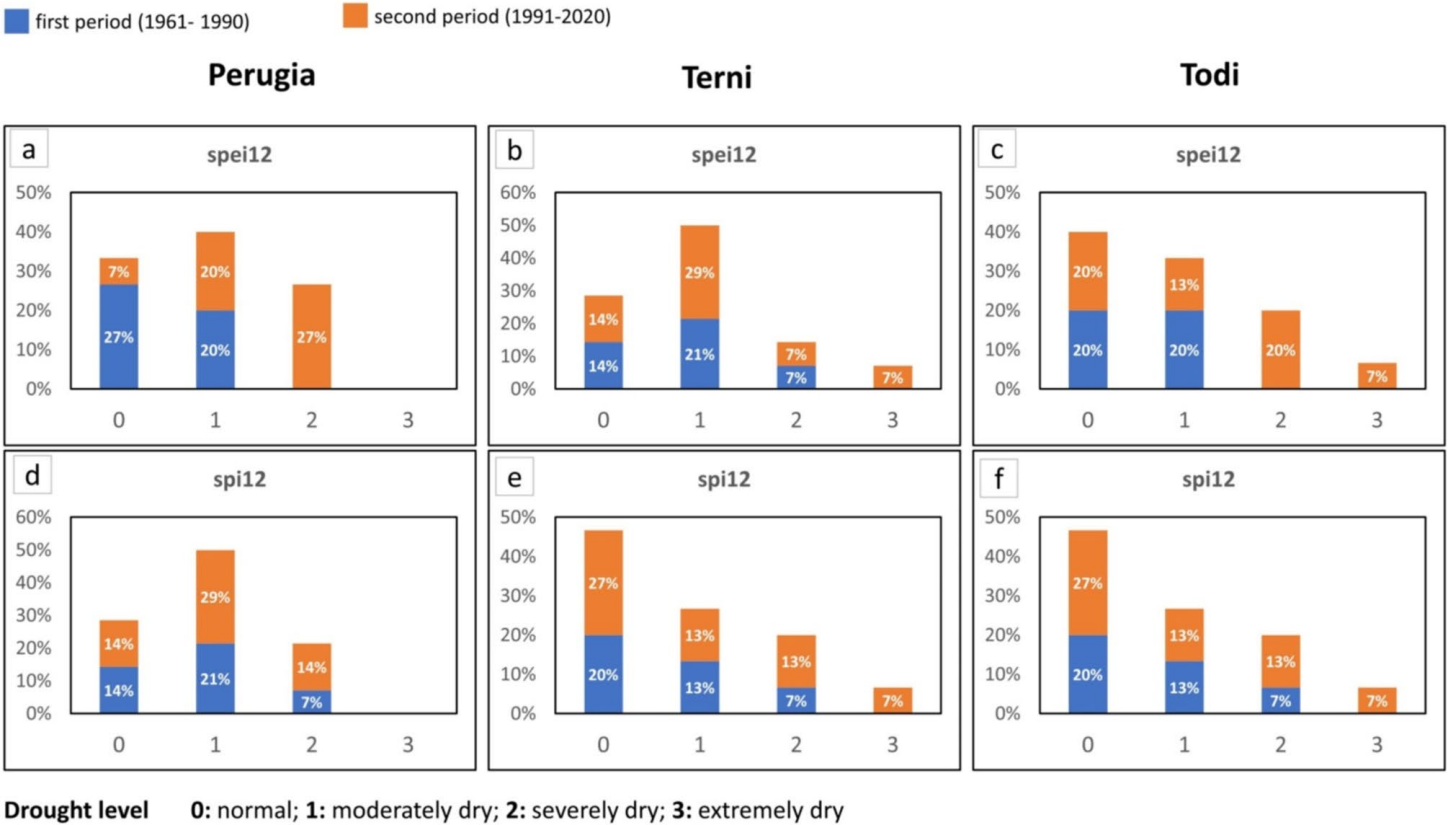
Distribution of drought levels (Svoboda et al. 2012) in the first and second periods (SPI 12, SPEI 12)

**Fig. 5:**
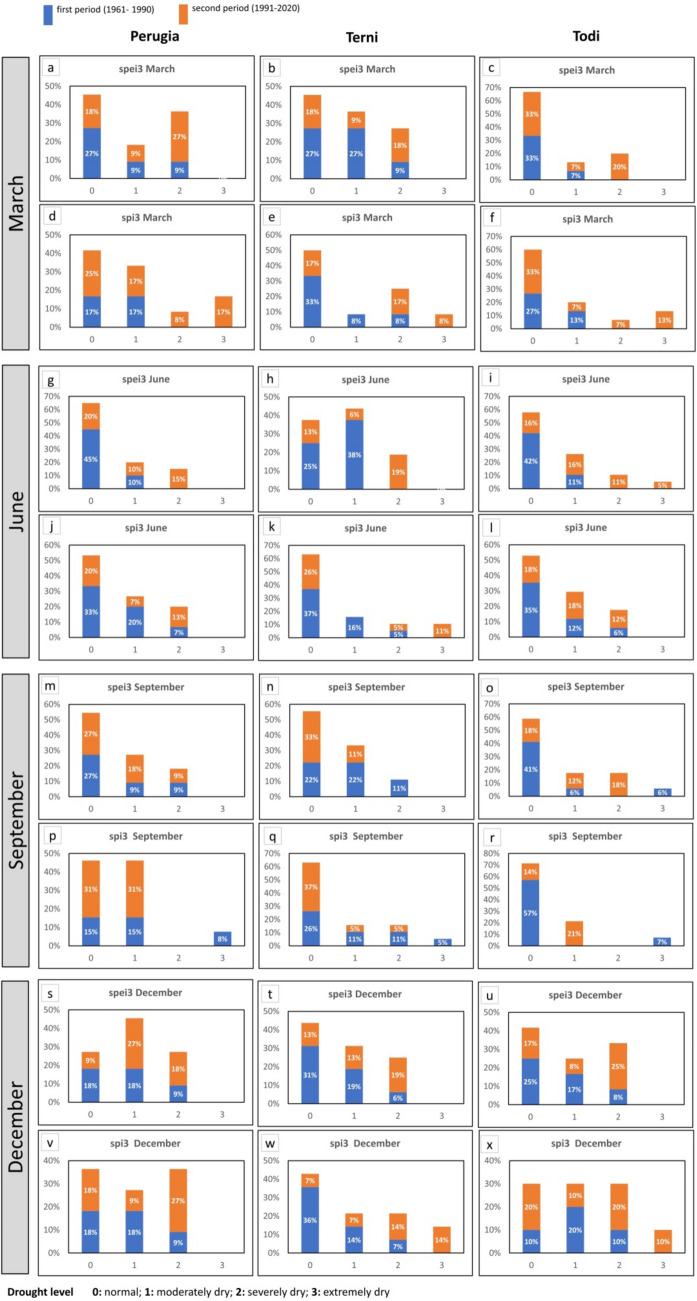
Distribution of drought levels (Svoboda et al. 2012) in the first and second periods (SPI 3, SPEI 3)

In Figures 4 and 5, the peak magnitude of run test analysis has been arranged in four classes, from 0 to 3, following the WMO guidelines in Table S1 (Svoboda et al. 2012): “0” identifies the normal level, “1” the moderately dry, “2” the severely dry and “3” the extremely dry level.

Generally, it is possible to observe: (i) the percentage of drought events, belonging to the most critical classes (second and third class) that fall in the first period is lower than in the second period and exceptions to the previous observation can be mainly found in peak values derived from the analysis of SPI; (ii) SPEI gives a percentage of critical events higher than SPI.

The first statement is valid with few exceptions: Terni, SPI 3 and SPEI 3 September (Figure 5, respectively chart n and q); Perugia, SPI 3 September, and Todi, SPI 3 September (Figure 5, respectively chart p, r).

Exceptions to the second statement can be found for Terni, SPEI 12, and SPI 12 (Figure 4, charts b and e); SPEI 3 and SPI 3 in March, September, and December (Figure 5, charts b, e, n, q, t, w). For Perugia, SPEI 3 and SPI 3 in December (Figure 5, charts s and v).

It is interesting to note that the general observations assumed from the first (boxplot charts) and the second (percentage of magnitude of the peaks in classes “2” and “3”) representation—respectively presented in Figure 3, Figure S11 (supplementary), Figure 4, and Figure 5—are similar, but the exceptions found are only partially superimposable. This is because the first is a statistical representation allowing us to consider and compare the median (average) value of a certain parameter, while the second one simply compares the percentages, for the whole events, that fall into the most critical classes.

Finally, it is possible to observe that only a few of the exceptions to the general observations described in this section (for example, ones regarding SPI 3 and SPEI 3 in September of Terni and Todi) correspond to the not significance of the trend of seasonal SPI/SPEI. Consequently, the results derived from significance tests (MK test, IST method) and the run test analysis can be considered related to each other but are not superimposable as a whole; then, they need to be taken into consideration together in the meteorological droughts studies.

### SPEI and SPI correlation

In this paragraph, the mathematical correlation based on the least squares linear regression between SPEI and SPI indices is considered, to detect its trend and, then, understand until it is possible to replace the SPEI index with SPI.

**Fig. 6:**
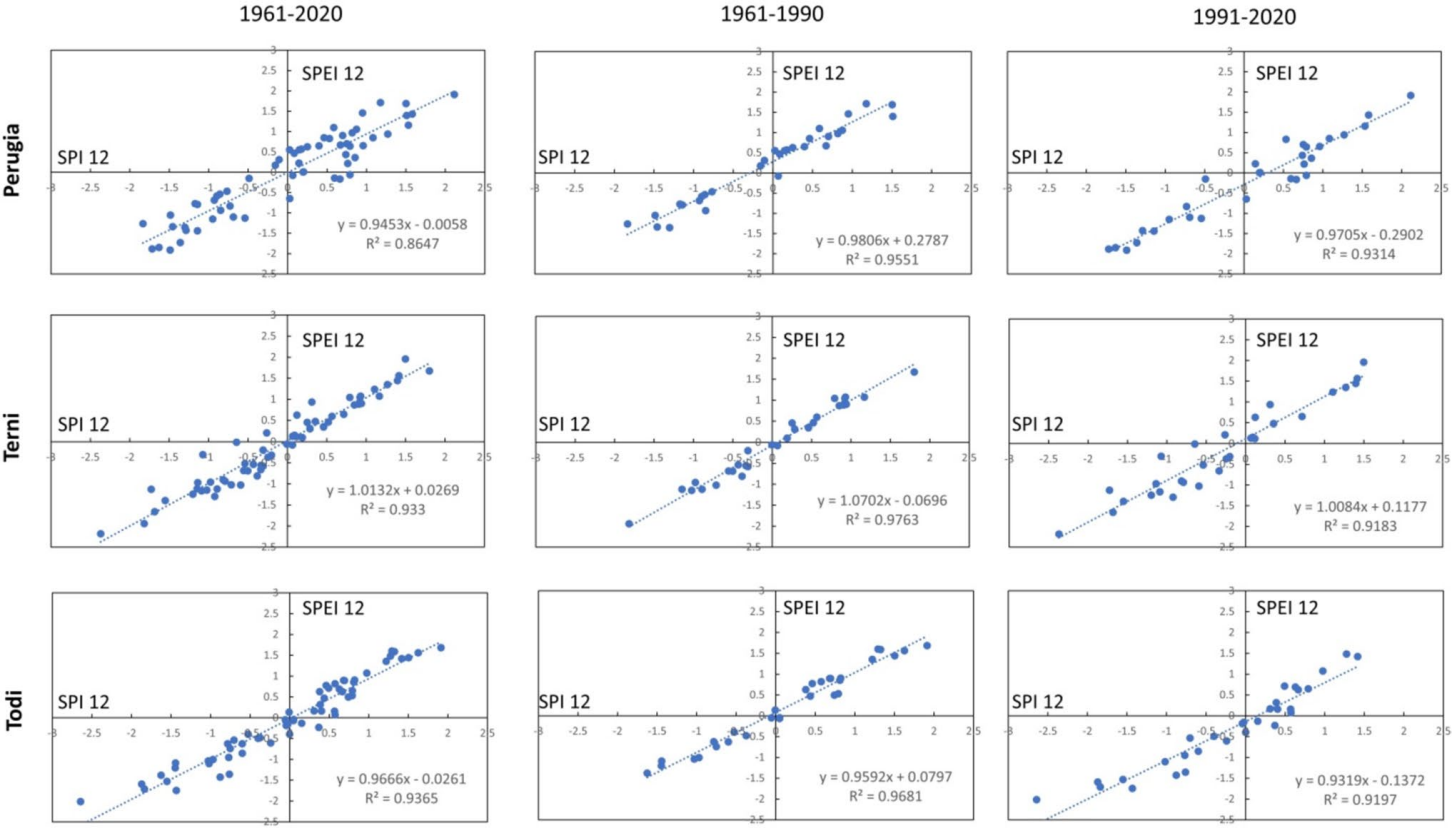
SPEI 12-SPI 12 (December) correlations: first column, 1961–2020; second column, 1961–1990; third column, 1961–2020

**Fig. 7:**
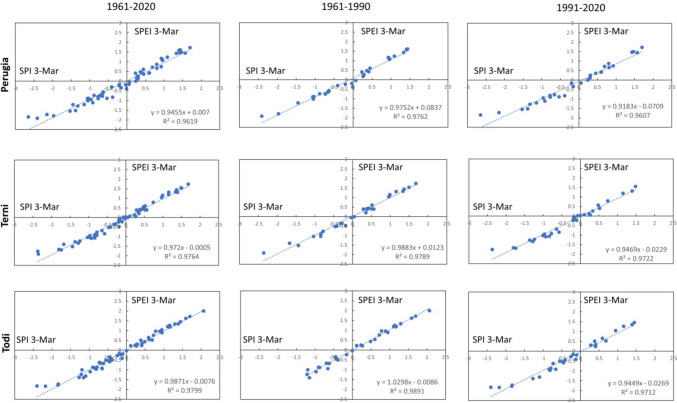
SPEI 3-SPI 3 (March) correlations: first column, 1961–2020; second column, 1961–1990; third column, 1961–2020

**Fig. 8:**
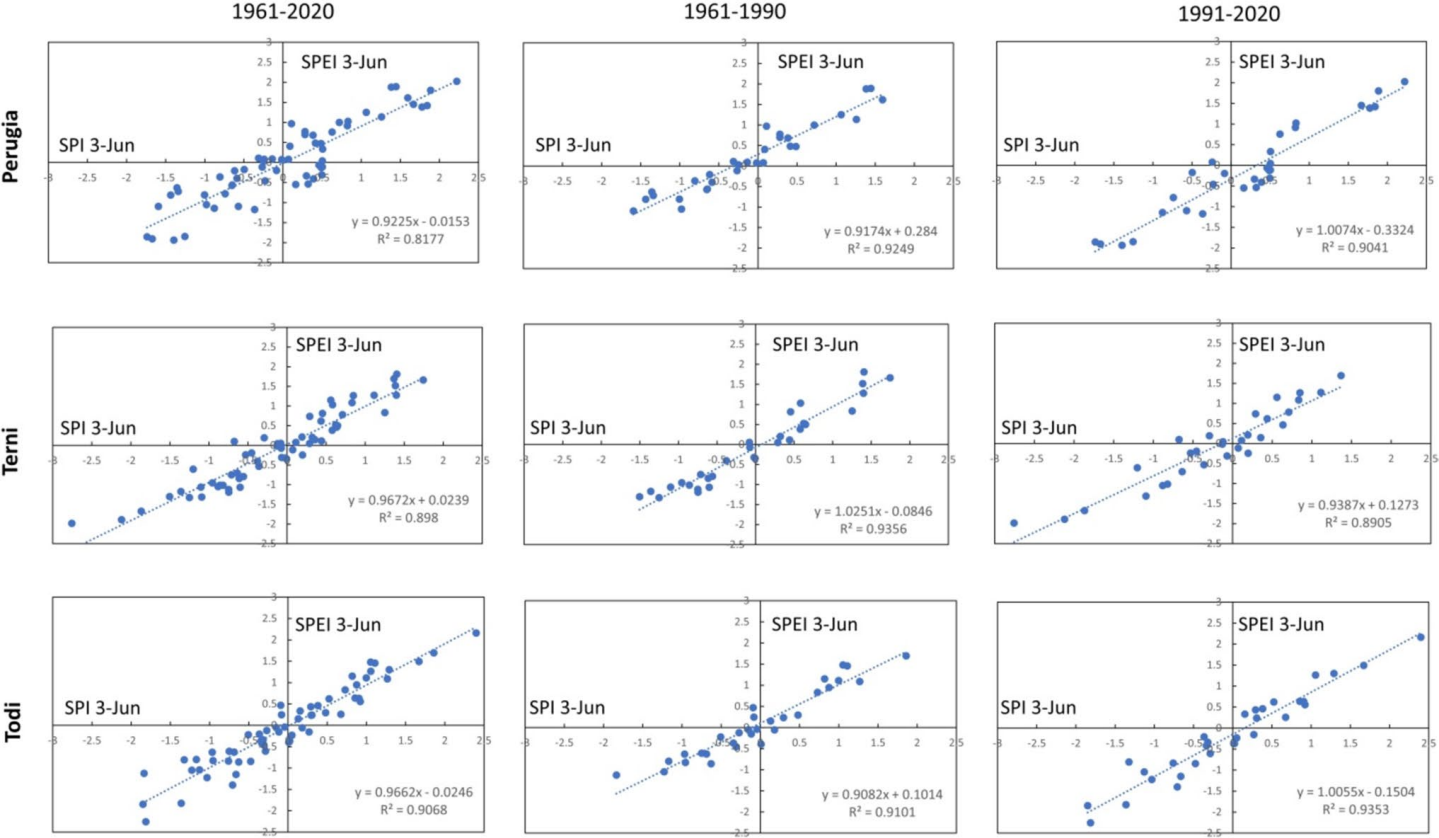
SPEI 3-SPI 3 (June) correlations: first column, 1961–2020; second column, 1961–1990; third column, 1961–2020

**Fig. 9:**
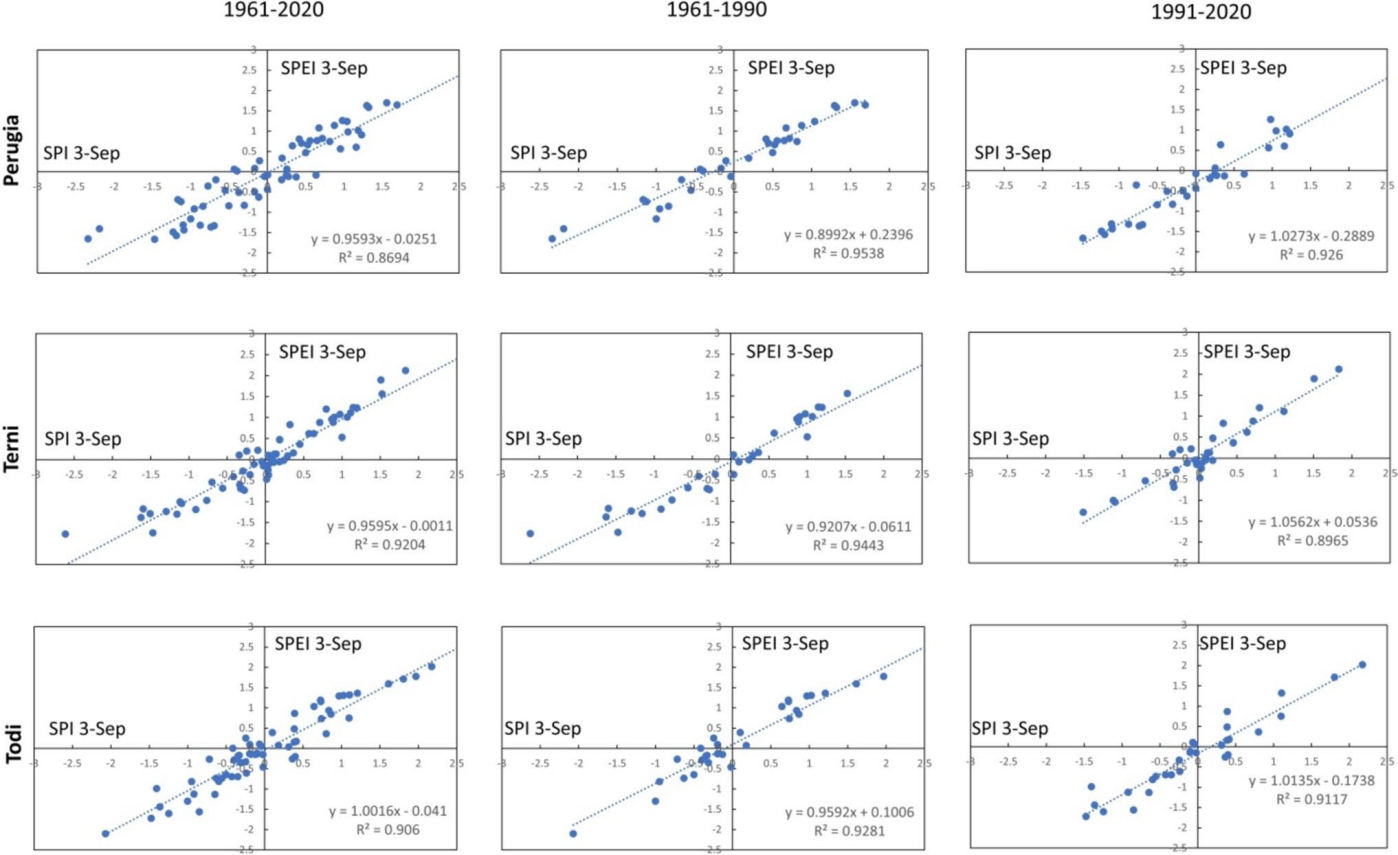
SPEI 3-SPI 3 (September) correlations: first column, 1961–2020; second column, 1961–1990; third column, 1961–2020

**Fig. 10:**
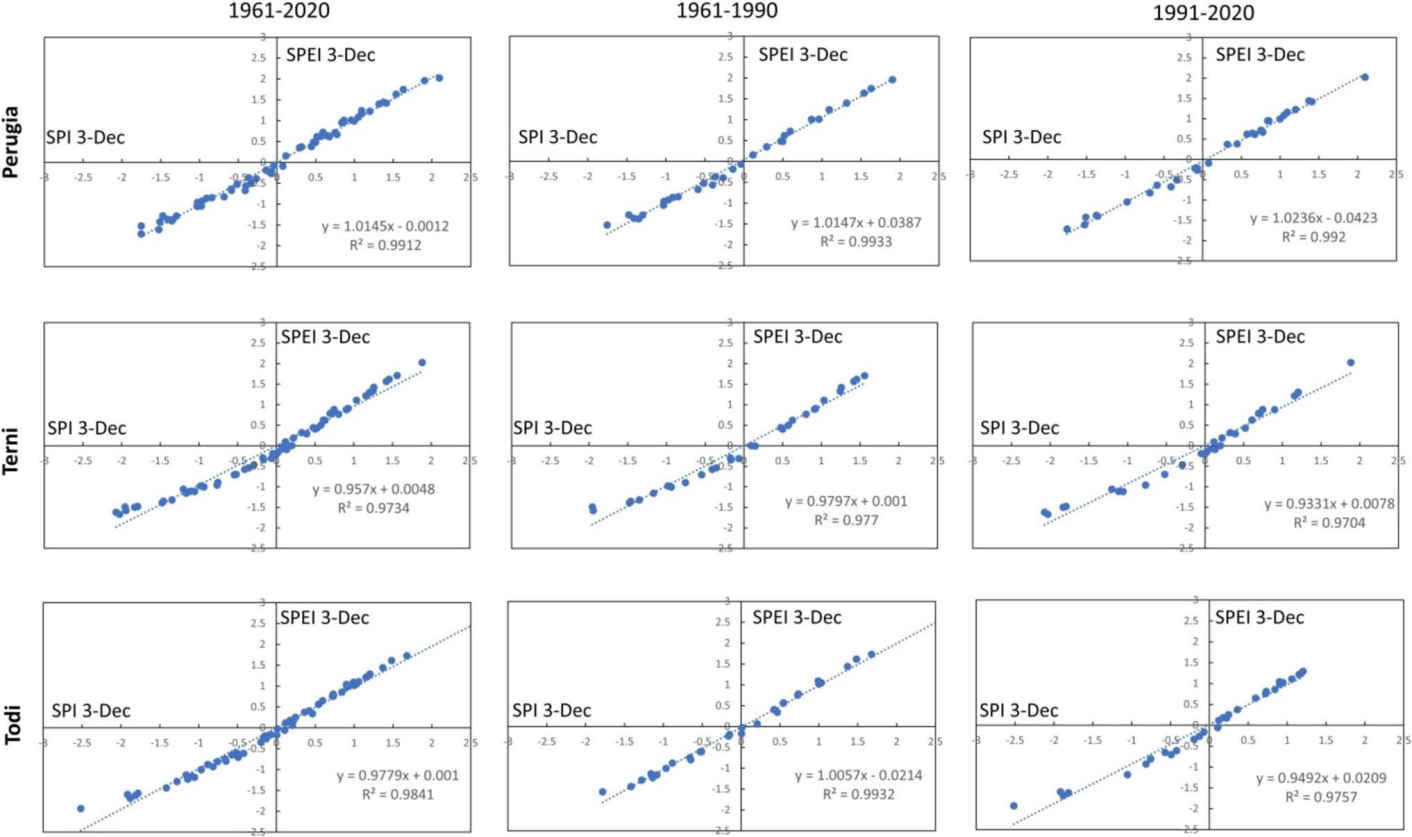
SPEI 3-SPI 3 (December) correlations: first column, 1961–2020; second column, 1961–1990; third column, 1961–2020

The linear relationships between SPI and SPEI for the different time scales are highlighted in Figs. 6, 7, 8, 9, and 10. In each graph is indicated the correspondent linear regression and the determination coefficient (${R}^{2}$), an indicator of how well the equation resulting from the regression analysis explains the relationship among the variables.

Actually, for SPEI 12-SPI 12 correlation (Fig. 6), it is possible to observe that the whole time series of Perugia presents the lowest value of ${R}^{2}$ (${R}^{2}$ = 0.8647). For a timescale of 3 months, significant correlations were observed in March (Fig. 7), at Todi, while the lowest value for the whole series was reached in June (${R}^{2}$ = 0.8177), at Perugia station (Fig. 8). Also, Perugia in September (Fig. 9) presents low ${R}^{2}$ values (0.869). In such a case, the worst approximation of linear regression is easily noticeable observing the higher spread of data points concerning the linear regression relationship and this is particularly evident when it is compared the first period with the second period. The highest correlations were observed for SPEI 3-SPI 3 December with many ${R}^{2}$ values above 0.99 (Fig. 10).

**Fig. 11:**
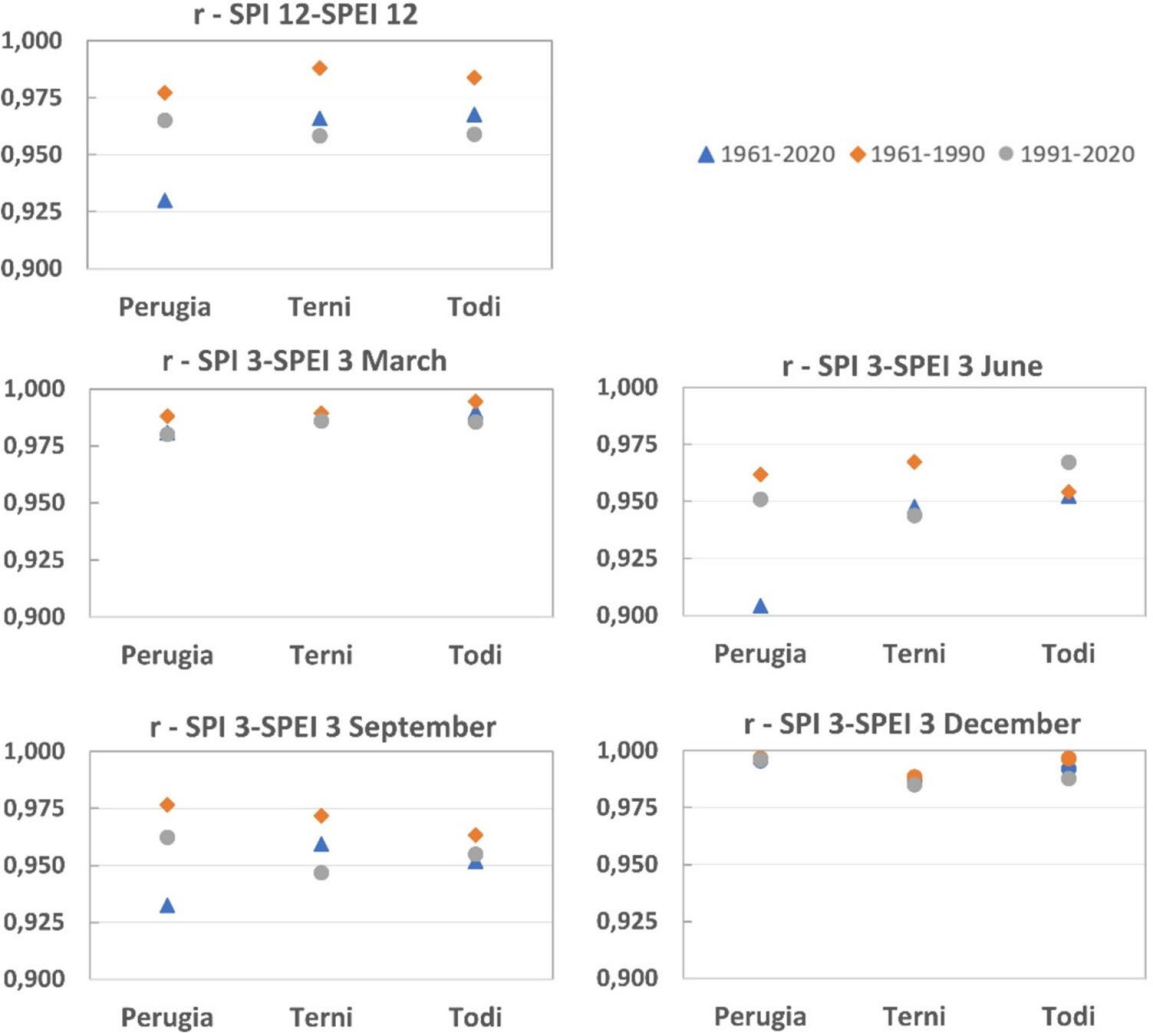
Graphs of Pearson coefficient for 12 and 3 (March, June, September, December) months. Periods, 1961–2020 (entire time series); 1961–1990 (first half of time series); 1991–2020 (second half of time series)

The Pearson correlation coefficient $r$ (see supplementary) is relatively high for all the time scales considered for all the stations (Table 4). Even for the *r* values, it is possible to observe a relevant difference between the first and the second periods (Fig. 11).

**Table 4:** Correlation SPI-SPEI: Pearson coefficient values (*r*)

SPI 12-SPEI 12, December			SPI 3-SPEI 3, March			SPI 3-SPEI 3, June			SPI 3-SPEI 3, September			SPI 3-SPEI 3, December		
r12	r12-1	r12-2	r3mar	r3mar-1	r3mar-2	r3jun	r3jun-1	r3jun-2	r3sep	r3sep-1	r3sep-2	r3dec	r3dec-1	r3dec-2
1961–2020	1961–1990	1991–2020	1961–2020	1961–1990	1991–2020	1961–2020	1961–1990	1991–2020	1961–2020	1961–1990	1991–2020	1961–2020	1961–1990	1991–2020
0.930	0.977	0.965	0.981	0.988	0.980	0.904	0.962	0.951	0.932	0.977	0.962	0.996	0.997	0.996
0.966	0.988	0.958	0.988	0.989	0.986	0.948	0.967	0.944	0.959	0.972	0.947	0.987	0.988	0.985
0.968	0.984	0.959	0.990	0.995	0.985	0.952	0.954	0.967	0.952	0.963	0.955	0.992	0.997	0.988

In particular, the correlation coefficient *r* is generally lower in the second period than in the first. This is always true for coefficient r12, with the highest difference between r12-1 and r12-2 for Terni and Todi (respectively, 3% and 2.5%). The difference between r3mar-1 and r3mar-2 is barely visible for Terni (0.3%), while it is more visible for the other stations. In June, the higher difference between r3jun-1 and r3jun-2 is placed in Terni (2.4%) while Todi represents an exception, and *r* becomes higher in the second period (−1.4%). In September, the highest difference was in Terni (2.6%) and the lowest in Todi (0.9%). In December, the differences between the first and second periods are very low and not significant.

### Possibilities of using the ERA 5 gridded dataset, Google Earth Engine, and Climate Engine

Monitoring of the climate conditions and forecasting of future trends relies on in situ measurements, satellite missions for Earth observation, and model estimates such as reanalysis products, which combine a recent numerical weather prediction (NWP) model with observational data (Hersbach et al. 2020). The suitability of the gridded datasets for completing missing gaps in the in situ measurements or to supplement other time series for computing SPI and SPEI is important to be tested. Generally, the Pearson correlation coefficient (*r*), coefficient of determination (*R*^2^), mean absolute error (MAE), root mean square error (RMSE), and other statistical methods are commonly used to compare modeled data with observations. Figure S12 shows an example of the observed time series of daily average temperature vs. ERA 5 reanalysis extracted data plotted for the year 1975 for the four meteorological stations. The linear regressions have very significant *R*^2^ values for all stations. In contrast, in an example plotted for the Terni station (Figure S13) for the years with severe droughts (from 1961 to 2020), the linear regressions for the observed time series of daily average precipitations vs. ERA 5 reanalysis extracted data showed reduced *R*^2^ values for all the years but increasing to the upper part of the interval (the highest *R*^2^ = 0.6 occurring for the years 2003 and 2007). For a finer assessment of the model, data agreement or disagreement, one can use indices that capture better the measured variance such as *d* (Index of agreement by Wilmott, 1981; see supplementary for details, Eq. 20).

In our study, the *d* values for the years that experienced severe droughts are presented in Table 5.

**Table 5:** Comparison of the observed temperature and precipitation at the meteorological station and ERA 5 reanalysis extracted data for the years recording severe droughts between 1961 and 2020 (*d*)

Year	Station	Temperature (2 m)	Total precipitation
1975	Todi	0.982	0.706
	Terni	0.948	0.671
	Perugia	0.989	0.638
1983	Todi	0.980	0.617
	Terni	0.963	0.678
	Perugia	0.990	0.558
2001	Todi	0.987	0.759
	Terni	0.991	0.759
	Perugia	0.990	0.779
2003	Todi	0.987	0.888
	Terni	0.994	0.848
	Perugia	0.996	0.832
2007	Todi	0.985	0.887
	Terni	0.970	0.796
	Perugia	0.977	0.820
2015	Todi	0.989	0.835
	Terni	0.976	0.847
	Perugia	0.981	0.860
2017	Todi	0.988	0.822
	Terni	0.977	0.871
	Perugia	0.984	0.842

Overall, the *d* values are very relevant for the temperature variable at all the stations and for all the selected years (the closer to 1, the more accurate the model is). The highest values were observed for the Perugia station for several years, while the lowest values occurred for the Terni station in the years 1975, 1983, and 2007. It can be concluded that there is a strong model-data agreement for temperature meaning that the ERA 5 reanalysis dataset could be considered for utilization with confidence in drought monitoring. On the other hand, regarding the precipitation assessment, *d* values have been slightly improved starting from 2003 (≥ 0.8) compared to the previous years. Overall, the modeled data has some relevance with reality in terms of the variation but more adjustments should be made to capture the real pattern, the maximum and minimum values of the observed time series as well as the simulated output. In the case of the total precipitation parameter from ERA 5, C3S points out that care should be taken when comparing model parameters with observations. This is because the observations are often local to a particular point in space and time, rather than representing averages over a model grid box. Recent studies (Bell et al. 2021; Lavers et al. 2022) show the importance of improving the precision of the precipitation data from ERA 5 to be used confidently across the globe in various simulations and forecasting. When comparing the average elevation of 5610 stations with the average ERA5 grid elevation, Lavers et al. (2022) found a difference of 56.6 m suggesting that the ERA 5 points generally have a higher elevation than the stations, which are predominantly situated in relatively low-lying areas. They also pointed out that the observed wet bias may be partly due to this elevation difference and potentially the orographic enhancement of precipitation. They also underlined the necessity of developing reanalyses with detailed orography and realistic land surface characteristics, which may reduce the representativeness issue, and by employing satellite gridded precipitation datasets or by using parametric approaches. In this regard, our future approach will consider new tests of agreement between data observed at ground-weather stations and satellite-derived datasets for improving precipitation time series (e.g., precipitation monthly and daily gridded data from 1979 to present derived from satellite measurements, https://cds.climate.copernicus.eu/cdsapp#!/dataset/satellite-precipitation; precipitation monthly and daily gridded data from 2000 to 2017 derived from satellite microwave observations, https://cds.climate.copernicus.eu/cdsapp#!/dataset/satellite-precipitation-microwave?tab=overview), or other reanalysis products or gridded datasets, such as MERRA-2 or EOBS data on a larger scale, and new data processing and data fusion applications. Furthermore, the use of complementary measurement systems including reliable networks of smart sensors (Casadei et al. 2021) for retrieving more data sources for the calculation of SPEI and SPI will be considered.

**Fig. 12:**
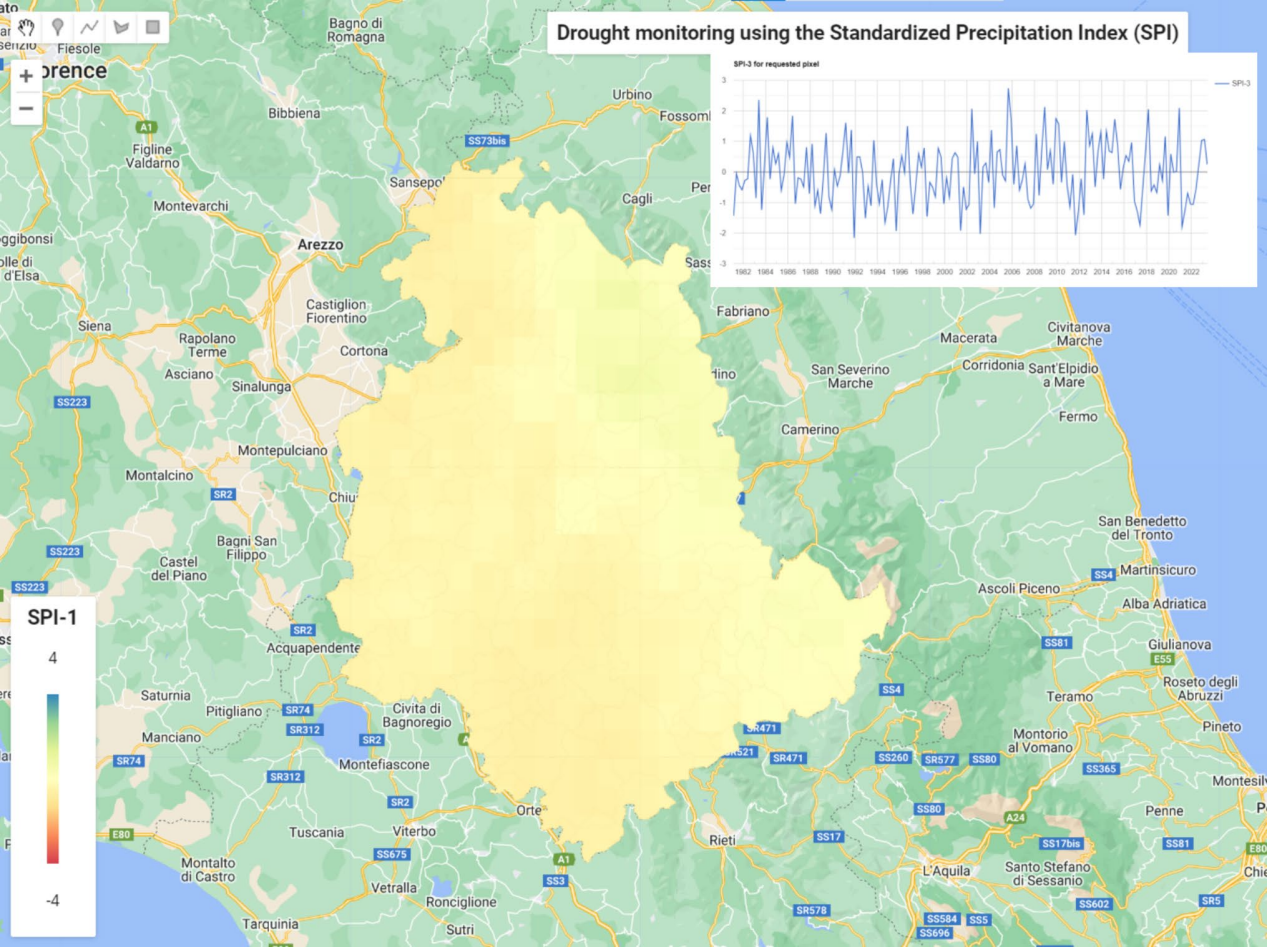
Drought assessment for Umbria region using the Google Earth Engine capabilities based on SPI 3 computed from CHIRPS; the graph shows the time series of SPI 3 from 1-Mar-1981 to 1-Jun-2023 for a selected pixel south from Todi

Another useful possibility is the utilization of Google Earth Engine (GEE) for computation of SPI based on the Climate Hazards Group InfraRed Precipitation with Station data (CHIRPS), which is a quasi-global rainfall dataset available from 1981 to the present. CHIRPS incorporates 0.05° resolution satellite imagery with in situ station data to create gridded rainfall time series for trend analysis and seasonal drought monitoring. The standard resolution is 5566 m. Figure 12 presents the map of Umbria with drought assessment with a pragmatic capability that is the computation of SPI for various periods and the visualization of time series for each selected pixel from the beginning of the dataset availability (since 1981). For the interpretation of SPI 3, the meteorological droughts (increasingly severe rainfall deficits) occurred when the value decreased below –1.0, while increasingly severe excess rainfall corresponded with SPI values above 1.0. On the other hand, SPI-1 may provide a good correlation between the soil moisture and the crop stress during the growing season as a short-term value. Many values of SPI 3 were estimated below − 1.0 and some of them even below − 2.0 (years 1992, 2003, and 2011) suggesting excessive dry conditions.

On the other hand, the Climate Engine application (ClimateEngine.org) can provide useful maps integrating valuable climate and hydrology datasets for visualization.

**Fig. 13:**
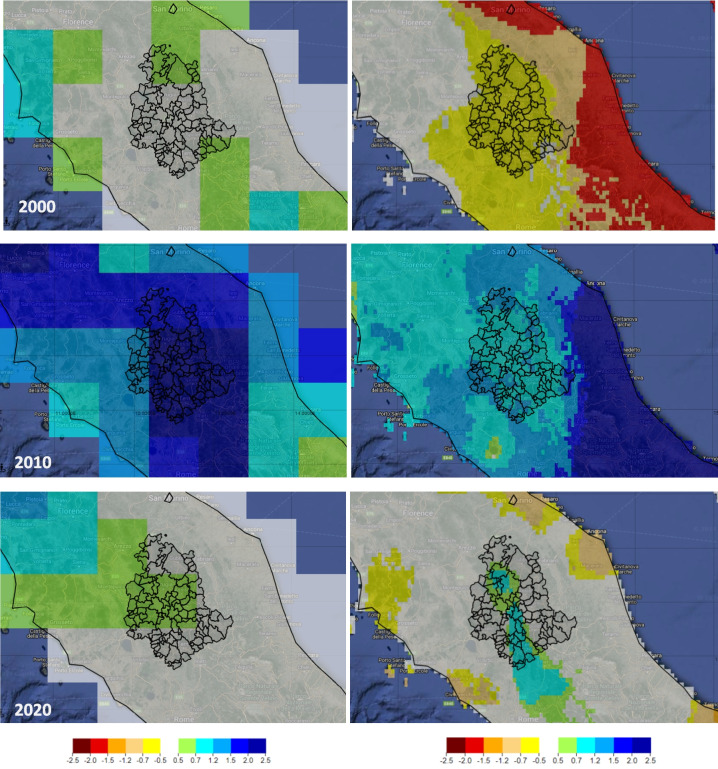
SPEI 12 maps for Umbria using MERRA2 (50 km)-left and Terraclimate (4 km)-right datasets for the years 2000, 2010, and 2020 (ClimateEngine.org)

Figure 13 presents the SPEI 12 maps for the Umbria region using MERRA2 (50 km) and Terraclimate (4 km) datasets for the years 2000, 2010, and 2020. In 2000, SPEI presented negative values for most of the Umbria region in both datasets matching the normal threshold, but lower in the case of Terraclimate (− 0.7 to − 0.5). The values modeled for the year 2010 were overestimated in the case of MERRA2 with pixels > 2 considered extremely wet, while the Terraclimate dataset provided values between 0.7 and 1.5. For 2020, positive values were retrieved in both datasets, showing a proper agreement. Consequently, the Climate Engine application can provide useful spatial assessments that can be adjusted for better performances based on SPI-SPEI correlation findings.

## Discussion

It is noteworthy to mention that the search in the Web of Science Core Collection using “SPEI-SPI correlation” in Topic yielded 192 results. Adding Italy in refining these results, it has been reduced to only 5 articles, all of them being recent works starting from 2019.

However, these studies are very different from our observations, since, although based on the analysis of SPEI and SPI, do not consider their reciprocal correlation but, mainly, associate SPEI and SPI with other indices or parameters. As an example, in Secci et al. (2021), the relationship between SGI, a statistical indicator of the groundwater severity, SPI and SPEI indices, modelled using historical data at stations in northern Tuscany, is used to estimate the future water levels; in the work of Vergni et al. (2021), SPI, SPEI, RDI and SDDI indices have been compared to stabilize their potential in detecting the impact of agricultural droughts in central Italy; Baronetti et al. (2024) study the correlation in northern Italy between two types of vegetation indices (NDVI and EVI) derived from MODIS images and SPI/SPEI indices, in order to detect the percentage of each type of vegetation undergoing drought stress; Russo et al. (2019), in order to prevent extreme weather events, consider the potential of SPEI/SPI indices of predicting extremely hot days/nights in summer in the Mediterranean region; finally, in Balacco et al. (2022), the correlations between SPI/SPEI and groundwater levels is studied in an aquifer of Salento region.

In this respect, our study completely differs from the ones listed above and it is centered on the analysis of the correlation of SPEI and SPI drought indices. In the “SPEI and SPI correlation” section, the regression analysis allows us to conclude that, in the Umbria region, it is not a proper choice to replace SPI with SPEI for drought monitoring: in fact, the marked growth of temperature in the last 30 years strongly modifies the value of SPEI respect to SPI and decreases the Pearson correlation coefficient between the two indices. This result is in agreement with the recent research of Lotfirad et al. (2021), who found that the correlation between SPEI and SPI is lower in regions of Iran with an arid climate.

The introductory analysis of trends based upon different types of tests (regression analysis, MK, and IST method) has put in evidence (“SPEI and SPI trends” section) that annual and seasonal SPEI is more significant than SPI in highlighting the increasing trend of droughts in all the stations taken into consideration.

Run theory (“Run test” section), in agreement with the negative trend of SPEI and SPI indices, underlines that the peak and intensity magnitude of drought events, in the period 1961–1990, is lower than in the period 1991–2020 and that SPEI provides higher values than SPI. Similarly, considering four classes of peak magnitude conforming to the WMO guidelines (Svoboda et al. 2012), the percentage of drought events in the most critical classes is higher in the second period, and SPEI gives a percentage of critical events higher than SPI.

In the “Possibilities of using the ERA 5 gridded dataset, Google Earth Engine, and Climate Engine” section, different types of resources for the evaluation of drought indices are considered. First of all, the CHIRPS dataset allows us to obtain the SPI index taking advantage of the potentialities of the GEE platform. Given the previous considerations, this tool becomes valuable when the results of correlation analysis confirm that the substitution of SPEI with SPI is possible. Then, elaborations based on the Climate Engine application are examined concluding that such tools can provide a quick and reliable evaluation of both SPEI and SPI.

In short, our study elucidates the intricate relationship between SPI and SPEI across various time intervals in the Umbria region (Italy). The correlation between these indices varies in time and space, influenced largely by climatic factors such as temperature variability and precipitation patterns. The differentiation between northern and southern areas of Umbria in terms of SPI-SPEI correlation is less significant, reflecting the quasi-homogenous climatic conditions within the region.

## Conclusions

In the next years, drought events are expected to rise in frequency, duration, and intensity.

The present study aimed at monitoring drought events in central Italy, Umbria region, by using SPI and SPEI indices obtained from a long time series of data (1961–2020), gauged and validated through the environmental monitoring systems. In particular, it intended to compare the effectiveness of SPI and SPEI indices verifying the trend and occurrence of droughts over historical time series and the possible correlation between the two indicators.

The results of the analysis based on monotonic trend tests and run theory indicate, for the annual and seasonal time scale, a general decreasing tendency of the indices, but with greater evidence for the SPEI, suggesting that SPEI can be considered more significant than SPI in highlighting the recurrence and characteristics of drought events.

Therefore, this study has focused on the possibility of estimating SPEI from SPI, since SPEI, which is also based on evapotranspiration estimation, requires a higher amount of data (both precipitation and temperature data) concerning SPI.

Nevertheless, in all the stations taken into consideration, the increase in the mean temperature negatively affects the quality of the correlation between the two indices and, consequently, in a future context of climate change, a drought analysis conducted using only the SPI index could be not reliable.

Given this, it would be necessary to exceed the prevailing use of the SPI to assess the drought phenomena and improve the environmental monitoring systems to have robust data both of rainfall and temperature in a high number of gauging stations that could be utilized also for a spatially distributed evaluation of SPEI index.

Indeed, future development could be to test the performance in computing the SPEI and SPI indices using large-scale products, such as gridded datasets of precipitation and temperature (Morsy et al. 2022), against the data collected at ground weather stations. ERA 5 and other datasets from C3S are well-established tools for climate monitoring that produce promising results for drought monitoring and assessment, especially in the areas with insufficient or without gauge stations, which are generally characterized by expensive operation and maintenance, point-scale, and time-demanding measurements.

Then, the findings of this study have important implications for drought monitoring and management and suggest that a one-size-fits-all approach to drought assessment may not be effective. Instead, a more region-specific approach, which employs also gridded datasets based on online tools and applications and takes into account the local climatic characteristics and the specific strengths of SPI and SPEI, is advisable. Additionally, incorporating other climatic factors such as wind patterns and soil humidity could further refine the understanding of drought phenomena. Another promising way is the application of advanced statistical and machine learning techniques to predict future drought conditions based on SPI and SPEI correlation findings performed using long time series from on-site meteorological measurements (Latifoğlu et al. 2024).

## Supplementary Information

Below is the link to the electronic supplementary material.

Supplementary file1 (PDF 3385 KB)

## Data Availability

Data will be made available on request from the corresponding author.
