# Supplementary material for: SPEI and SPI correlation in the study of drought phenomena in Umbria region (central Italy)
Source: Environ Sci Pollut Res Int. 2024 Dec 15;32(1):168–88. doi: 10.1007/s11356-024-35740-2 (PMC11717805; doi:10.1007/s11356-024-35740-2)
Supplement: Supplementary file 1 — Supplementary file1 (PDF 3385 KB) [file 11356_2024_35740_MOESM1_ESM.pdf]

SPEI and SPI correlation in the study of drought phenomena in Umbria region (Central Italy)

Sara Venturi[1], Daniel Dunea[2,*], Elena Mateescu[3], Ana Virsta[4], Nicolae Petrescu[2], Stefano Casadei[1]

[1]Department of Civil and Environmental Engineering, University of Perugia, Via Duranti 93 06125 Perugia, Italy

[2]Valahia University of Targoviste, Aleea Sinaia no.13, Targoviste 130004, Romania

[3]National Administration of Meteorology, Șoseaua București-Ploiești 97, Bucharest 013686, Romania,

[4]University of Agronomic Sciences and Veterinary Medicine of Bucharest, 59 Marasti Blvd., District 1, Bucharest 011464, Romania

**Corresponding author e-mail: dan.dunea@valahia.ro*

Table S1: SPI (SPEI) classes with their corresponding intervals and theoretical occurrence probabilities (according to WMO's SPI User Guide; Svoboda et al., 2012).

SPI (SPEI) interval	SPI (SPEI) class	Occurrence probability (%)	
SPI ≥ 2	extremely wet	2.3	1 in 50yrs.
2 > SPI ≥ 1.5	severely wet	4.4	1 in 20yrs.
1.5 > SPI ≥ 1	moderately wet	9.2	1 in 10yrs.
1 > SPI > −1	normal	68.2	1 in 3years
−1 ≥ SPI > −1.5	moderately dry	9.2	1 in 10yrs.
−1.5 ≥ SPI > −2	severely dry	4.4	1 in 20yrs.
SPI ≤ −2	extremely dry	2.3	1 in 50yrs.

S.1.1 Computation of Standardized Precipitation Evapotranspiration Index (SPEI)

In the SPEI computation, for the i- month, the difference D_i (water balance) between the precipitation P_i and potential evapotranspiration PET_i is considered as input:

$$D_i = P_i - PET_i \quad (1)$$

where D_i in eq. (1) provides a simple measure of the water surplus or deficit (Serrano et al. 2010).

The calculated D_i values have been aggregated at different time scales (3 and 12 months).

According to Yang et al. (2021), the Penman method is the most physical-based and reliable method to calculate PET. However, the required data (air temperature, relative humidity, wind, net radiation), make it to use more challenging than other techniques.

In this study, evapotranspiration is calculated using the Hargraves-Samani formula (Hargraves et al., 2003; Beguería et al., 2014):

$$PET_i = 0.0023 \cdot (T_{i,mean} + 17.8) \cdot (T_{i,max} - T_{i,min})^{0.5} \cdot R_{i,a} \cdot 0.408 \tag{2}$$

where PET_i is expressed in mm; $T_{i,mean}$, $T_{i,max}$, $Ti,_{min}$ are, respectively, the average, maximum, and minimum temperature in °C; $R_{i,a}$ is the extra-terrestrial radiation expressed in MJ m^{-2}, calculated as a function of the latitude and the month of the year.

The three parameters log-logistic distribution is chosen as fitting distribution. The probability density (PDF) of a variable x is expressed as:

$$f(x) = \frac{\beta}{\alpha}\left(\frac{x-\gamma}{\alpha}\right)^{\beta-1}\left[1+\left(\frac{x-\gamma}{\alpha}\right)^{\beta}\right]^{-2} \tag{3}$$

where α, β , and γ are, respectively, the scale, shape, and origin parameters.

Parameters of the PDF can be calculated as a function of L-moments of D_i series, subsequentially obtained from probability-weighted moments (PWMs), indicated with w_0, w_1, w_2 (Singh et al., 1993):

$$\beta = \frac{2w_1 - w_0}{6w_1 - w_0 - 6w_2} \quad \alpha = \frac{(w_0 - 2w_1)\beta}{\Gamma(\beta)\left(1+\frac{1}{\beta}\right)\Gamma(\beta)\left(1-\frac{1}{\beta}\right)}$$
$$\gamma = w_0 - \alpha\,\Gamma(\beta)\left(1+\frac{1}{\beta}\right)\Gamma(\beta)\left(1-\frac{1}{\beta}\right) \tag{4}$$

where $\Gamma(.)$ is the Gamma function of β.

PWMs are estimated as in Serrano et al.et al. (2010), using the *plotting-position* approach:

$$w_s = \frac{1}{n}\sum_{i=1}^{n}(1-F_i)^s D_i \qquad s = 0,1,2 \tag{5}$$

where n is the sample size and $F_i = \frac{i-0.35}{n}$ is a frequency estimator (Hosking, 1986).

The cumulative distribution function (probability of exceeding a determined value of D_i) is:

$$F(x) = \left[1+\left(\frac{\alpha}{x-\gamma}\right)^{\beta}\right]^{-1} \tag{6}$$

Then, SPEI can be easily obtained as the standardized values of $F(x)$ using the approximated formulas (Abramowitz and Stegun, 1965):

$$SPEI = \begin{cases} t - \dfrac{c_0 + c_1 t + c_2 t^2}{1 + d_1 t + d_2 t^2 + d_3 t^3} & t = \sqrt{-2\, ln\, P_r} \quad P_r \leq 0.5 \\ -\left(t - \dfrac{c_0 + c_1 t + c_2 t^2}{1 + d_1 t + d_2 t^2 + d_3 t^3}\right) & t = \sqrt{-2\, ln\,(1 - P_r)} \quad P_r > 0.5 \end{cases} \tag{7}$$

where $P_r = 1 - F(x)$ and represents the probability of exceeding a certain value of *D*. The values of the coefficients c and d are:

$$c_0 = 2.515517, c_1 = 0.802853, c_2 = 0.010328, d_1 = 1.432788, d_2 = 0.189269, d_3 = 0.001308$$

S.1.2 Computation of Standardized Precipitation Index (SPI)

SPI index is computed by fitting monthly precipitation data to a Gamma PDF (Edwards et al., 1997):

$$g(x) = \frac{1}{\beta^\alpha \Gamma(\alpha)} x^{\alpha-1} e^{-\frac{x}{\beta}} \tag{8}$$

where α and β are, respectively, the shape and scale parameters. $\Gamma(.)$ is the Gamma function.

Although recent studies (e.g., Pieper et al., 2020) have suggested the use of alternative distributions for the estimation of the SPI index, the choice of Gamma distribution remains the most common in literature (Wu et al., 2007). Then, the cumulative probability assumes the following form:

$$G(x) = \int_0^x g(t)dt \tag{9}$$

Since $\Gamma(\alpha)$ is undefined for x=0 and a precipitation distribution can present zero values, Thom (1966) proposed that the cumulative probability $G(x)$ could be alternatively defined as:

$$H(x) = q + (1 - q)\, G(x) \tag{10}$$

where q is the probability of a zero in a precipitation time series and is estimated as $q = \frac{m}{n}$, with m equal to the number of zeros and n to the number of observations in the sample.

S.1.3 Mann-Kendall (MK) computation

The MK statistic S is estimated according to the following formula:

$$S = \sum_{k=1}^{n-1} \sum_{j=k+1}^{n} sgn(x_j - x_k) \tag{11}$$

where n is the specimen size, sgn is the signum function extracting the sign of the difference between series terms: x_j and x_k (k=1, 2, …, n-1; j=k+1, k+2, …, n). The signum function assumes the value:

$$sgn(x_j - x_k) = \{+1 \quad if\ (x_j - x_k) > 0 \quad 0 \quad if\ (x_j - x_k) = 0 \quad -1 \quad if\ (x_j - x_k) < 0 \tag{12}$$

In the study of Hamed (2008), it is observed that only the observations rank influences the value of S, instead of their actual values, following a distribution-free test statistic.

The mean of S is $E(S) = 0$ and the variance is:

$$Var(S) = \frac{n(n-1)(2n+5)}{18} \tag{13}$$

The effect of tied ranks (or equal observations) in the data reduces the value of the variance of S:

$$Var(S) = \frac{n(n-1)(2n+5)}{18 - \frac{\sum_{j-1}^{m} t_j(t_j-1)(2t_j+5)}{18}} \tag{14}$$

where m is the number of groups of tied ranks, each with t_j tied observations.

The comparison between the standardized variable Z_{MK} in eq. (15) and the standard normal variable at a given significance level α (in this study equal to 0.05) allows testing the significance of the trend.

$$Z_{MK} = \begin{cases} \sqrt{\frac{S-1}{Var(S)}} & if\ S > 0 \\ 0 & if\ S = 0 \\ \sqrt{\frac{S+1}{Var(S)}} & if\ S < 0 \end{cases} \tag{15}$$

A positive Z_{MK} value indicates an upward trend and a negative value corresponds to a downward trend. But the trend is statistically significant only if $|Z_{MK}|$ > ($Z_{1-\frac{\alpha}{2}}$)=1.96, corresponding to the value of the standard normal variable for the chosen significance level.

S.1.4 Innovative-Sen trend (IST) computation

The test is applied by splitting time-series data into two subsets and then arranged in ascending order in a cartesian coordinate system. The 1st half series is plotted on the x-axis (horizontal axis), and 2nd half series is plotted on the y-axis (vertical axis). An upward or downward trend exists in the time series if data fall, respectively, above or below the 1:1 line (Figure S1). If the scatter points of the data appear on the 45° straight line or close to this line, it means that there is no significant trend (no-trend time series) (Sen, 2012, Dabanlı et al., 2016).

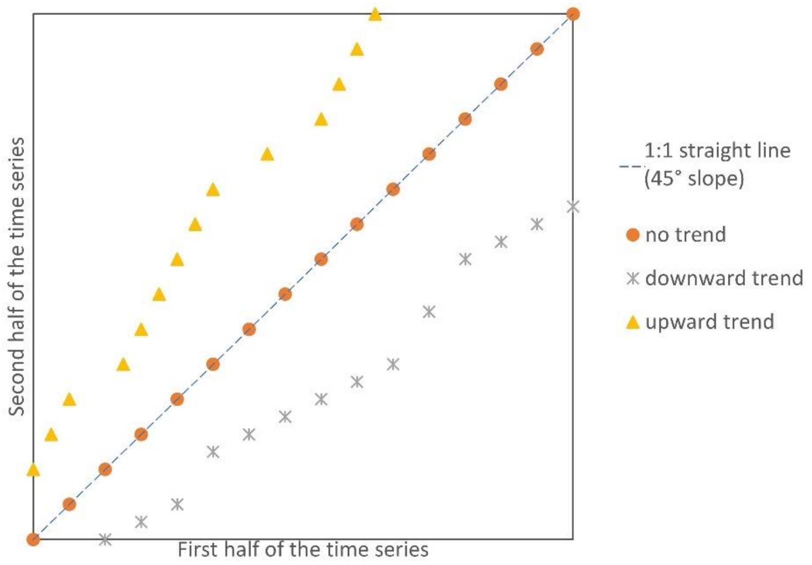

Figure S1. Decreasing, increasing trends and trendless regions.

However, in some cases, the visual inspection of the two subsets in a cartesian coordinate system does not guarantee an immediate interpretation, for example, when the points cross the 45° straight line (Dabanlı et al., 2016).

In 2017, Sen overcame the qualitative approach and first presented and formulated a procedure based on the evaluation of the trend slope (s) of the time series. The trend slope s of the time series is:

$$s = \frac{2 \cdot \left(\underline{y_2} - \underline{y_1}\right)}{n} \tag{16}$$

where $\underline{y_1}$ and $\underline{y_2}$ are the coordinates of the centroid point of the two time series and n is the size of the sample.

In Figure S2, only for demonstration purposes, the 1st and 2nd half ordered time series of the precipitation in a sample station (Terni station) are plotted in a cartesian system. In the sample, $= 60$, $\underline{y_1} = 910.09$ mm and $\underline{y_2} = 847.52$ mm, then $s = -2.08$. The grey line (trend line) indicates the trend that data would have if they were characterized by the same centroid point but by a linear tendency. The vertical distance between the 1:1 straight line and the trend line is equal to $910.09 - 847.52$ mm.

In Sen (2017), it is demonstrated that the variance ${\sigma_s}^2$ of the slope parameter s is a function of, σ^2, the standard deviation of entire series data, and of $\rho_{\underline{y_1}\underline{y_2}}$, the correlation coefficient between the two sorted half-series:

$${\sigma_s}^2 = \frac{8}{n^2}\frac{\sigma^2}{n}\left(1 - \rho_{\underline{y_1}\underline{y_2}}\right) \tag{17}$$

The upper/lower confidence limits (CL) of the trend slope s at the significance level (α) is:

$$CL_{1-\alpha} = 0 \pm \sigma_s s_{cri} \tag{18}$$

where s_{cri} is the confidence limit of a standard normal distribution ($\mu = 0, \sigma = 1$) at α percent of significance level (α=0.05, s_{cri}=1.96). If s falls outside the confidence limit in eq. (18), a trend exists and it is increasing/decreasing if s is, respectively, positive/negative.

A positive/negative value of s indicates an increasing/decreasing trend in the time series.

In Figure S2, the standard deviation of the sample series is $\sigma = 171.67$ and the correlation coefficient between the two half-series is $\rho_{\underline{y}_1\underline{y}_2}$ =0.92, then the standard deviation of the trend slope is $\sigma_s = 0.294$. The confidence limits $CL_{1-\alpha} = \pm 0.294 \cdot 1.96 = \pm 0.57$: the trend slope s falls outside the confidence interval, then a monotonic decreasing trend occurs at a significance level α=0.05.

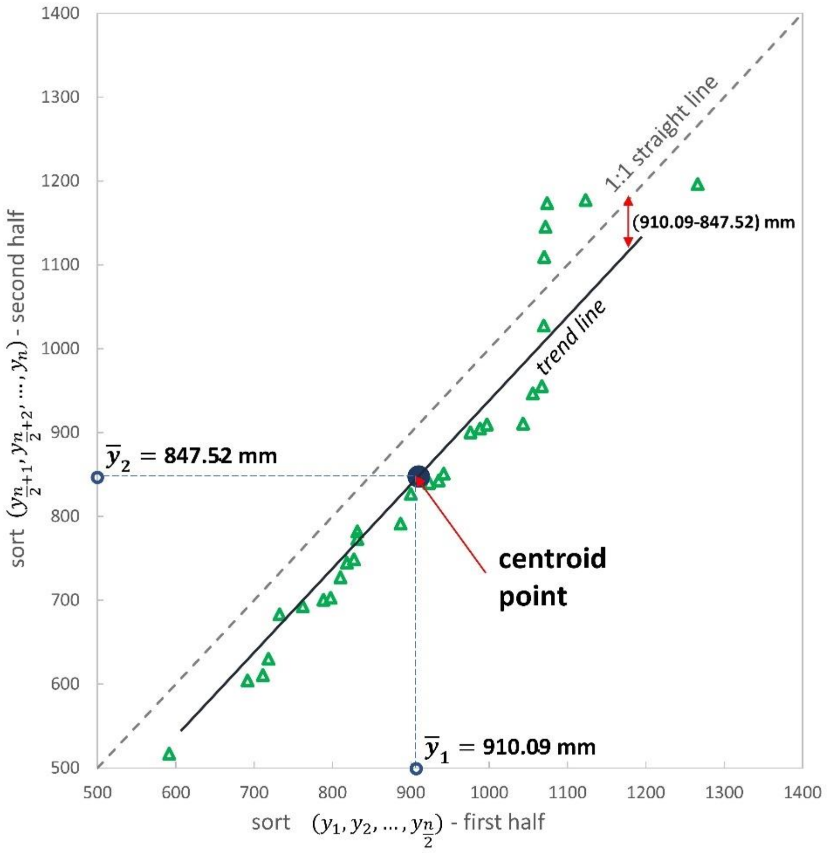

Figure S2. Precipitation time-series of Terni station: trend slope calculation using IST method.

S.1.5 Study Area

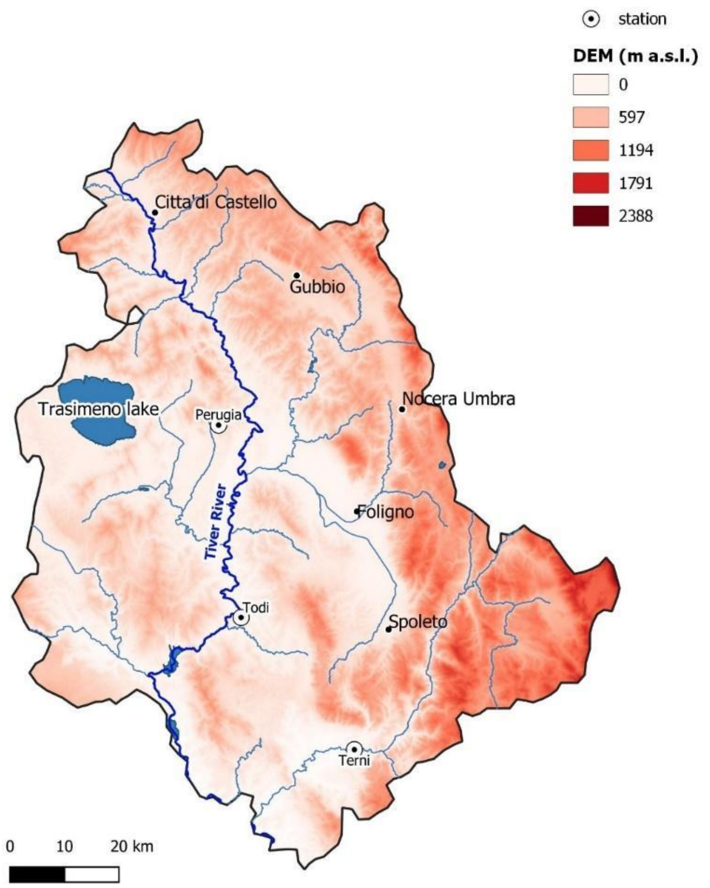

Figure S3. Morphology of the Umbria region (Italy) and stations used in this study (left). The background Digital Elevation Model (10 m spatial resolution) is derived by TINITALY DEM (https://doi.org/10.13127/TINITALY/1.0).

Precipitation and temperature data source

Table S2: Rainfall- temperature weather stations: geographic position, periods of data availability.

Station		WGS 84 (EPSG: 4326) Longitude (°)	WGS 84 (EPSG: 4326) Latitude (°)	Altitude (m a.s.l.)	Rain gauge	Availability		Air thermometer	Availability	
Perugia	Perugia Fontivegge	12.376	43.106	345	✔	1995-2022	Daily and monthly average, minimum and maximum rainfall [mm]; Extreme rainfall (24 h, 48 h) [mm]	✔	1994-2022	Daily and monthly average, minimum and maximum Temperature [°C];
	Perugia Santa Giuliana	12.387	43.105	417	✔	1988-2022		✔	1990-2022	
	Perugia S. Pietro	12.396	43.101	439	✔	1946-1988		✔	1951-1987	
Terni		12.65	42.56	130	✔	2019-2022		✔	1951-2022	
Todi		12.41	42.786	331	✔	1919-2022		✔	1919-2022	

Precipitation and temperature: evaluation and comparison of trend

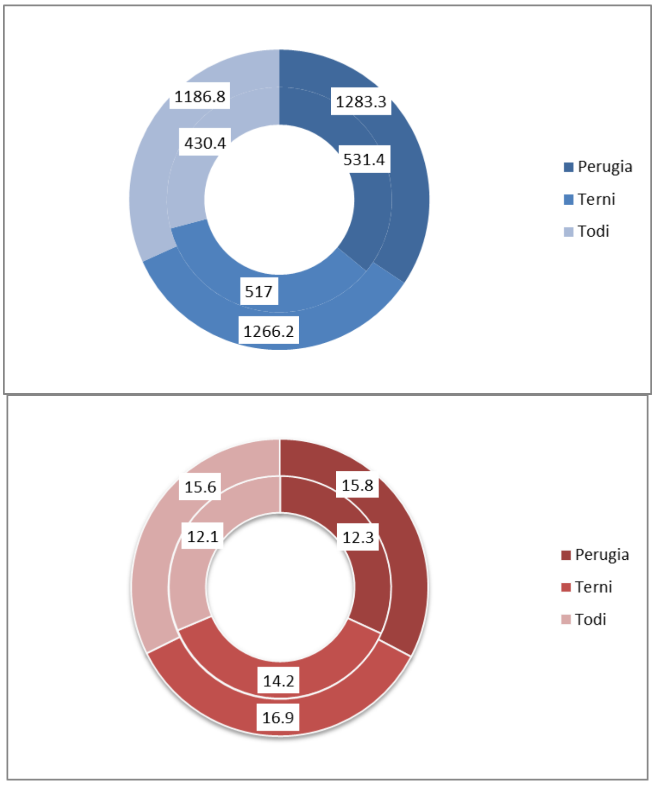

Figure S4. Minimum and maximum averages (a) Radar chart of annual cumulative precipitation; (b) Radar chart of annual average temperature.

S.1.6 SPEI and SPI trend

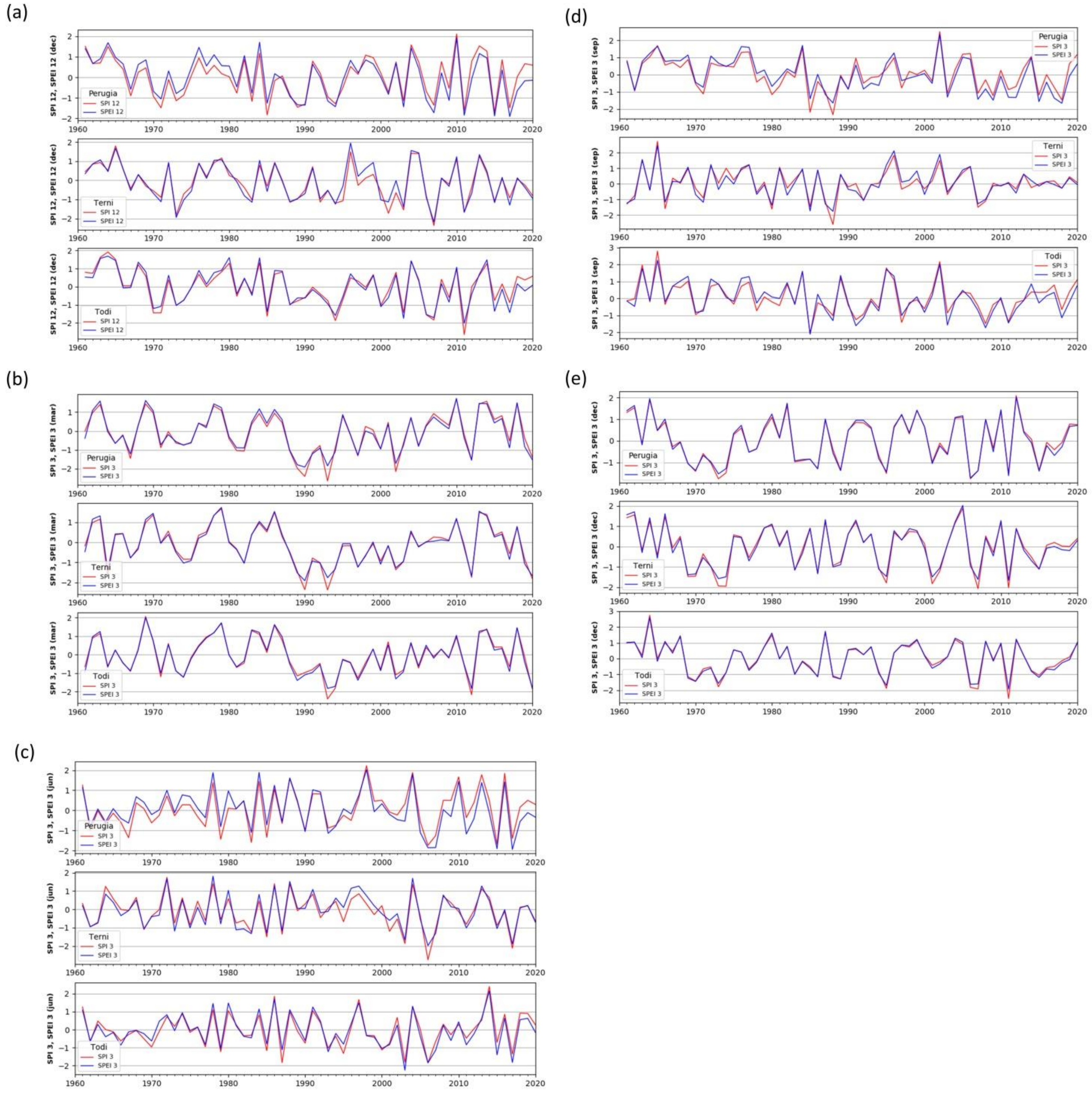

Figure S5. Stations: Perugia, Terni, Todi. SPI 12, SPEI 12 (a). SPI 3, SPEI 3 (b-March, c-June, d-September, e-December).

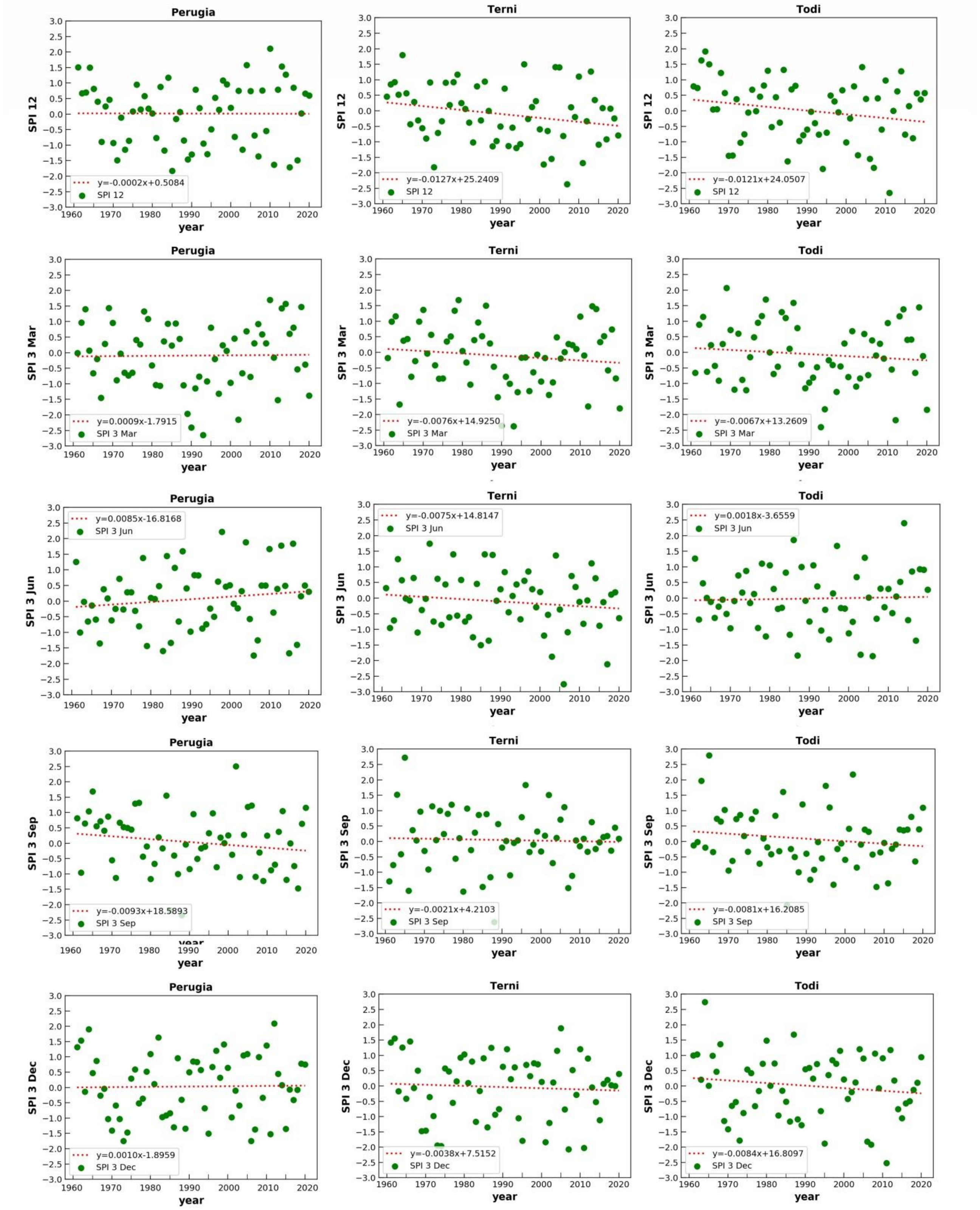

Figure S6. Linear regression – SPI 12 and seasonal SPI 3

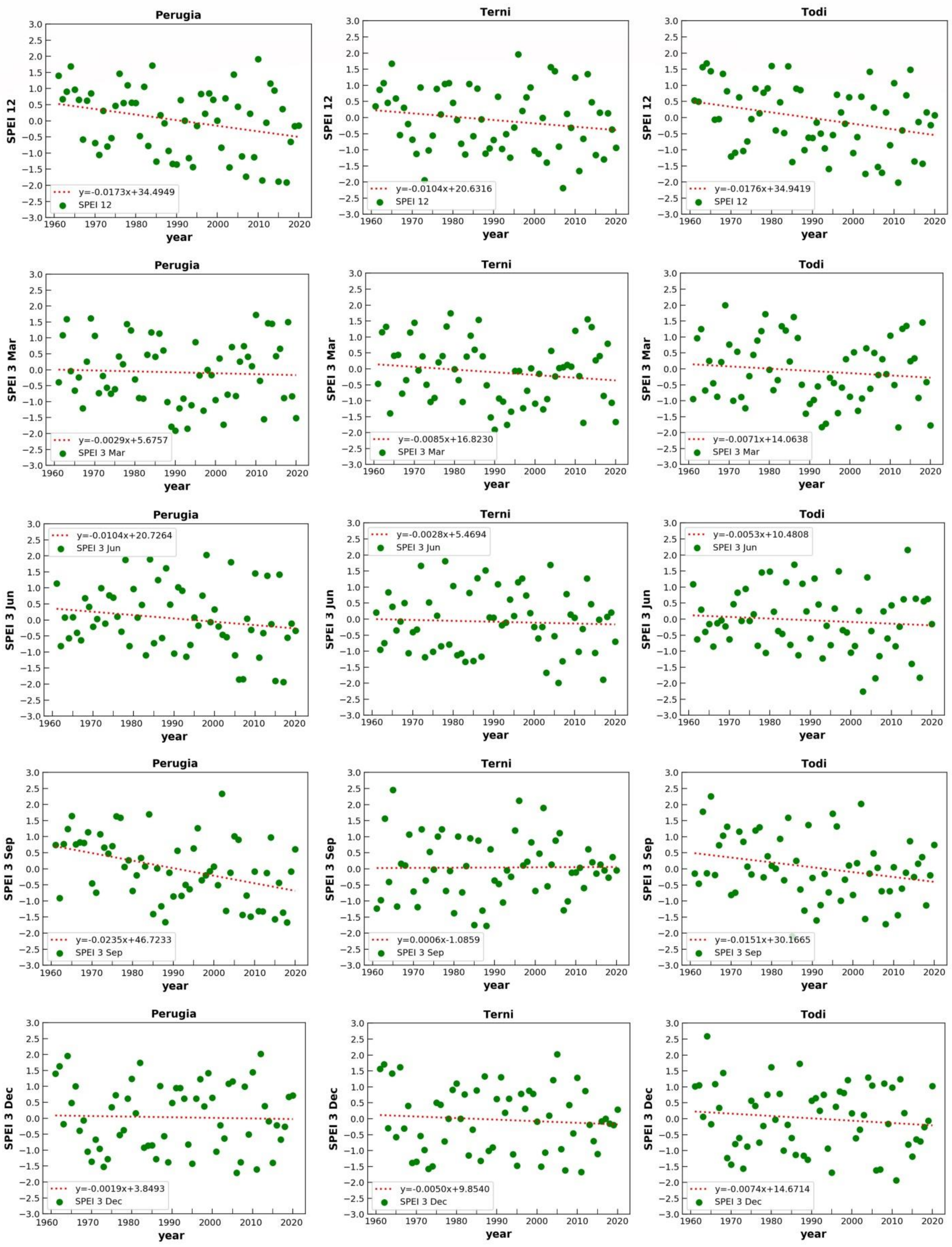

Figure S7. Linear regression – SPEI 12 and seasonal SPEI 3

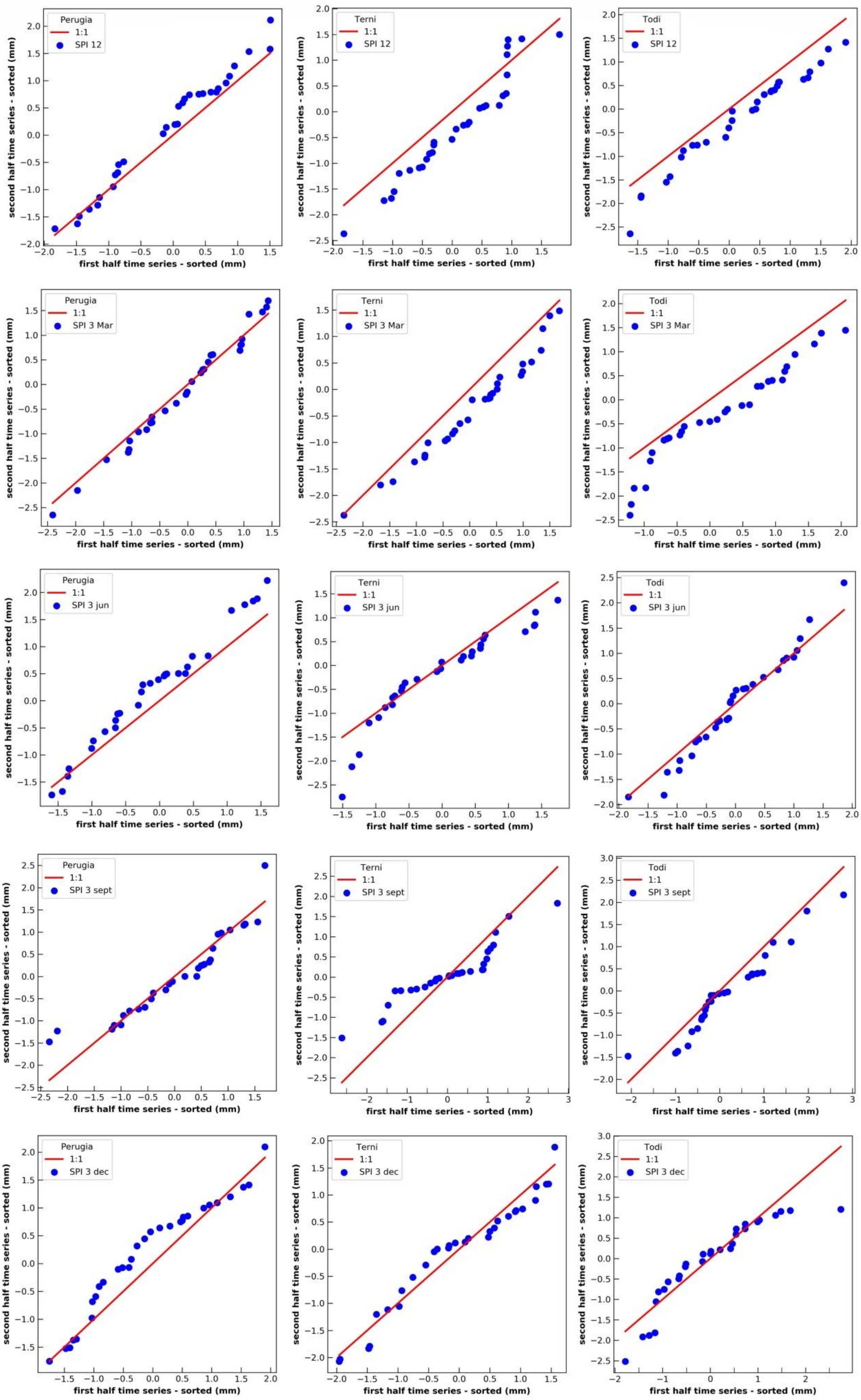

Figure S8. IST method - SPI 12 and seasonal SPI 3

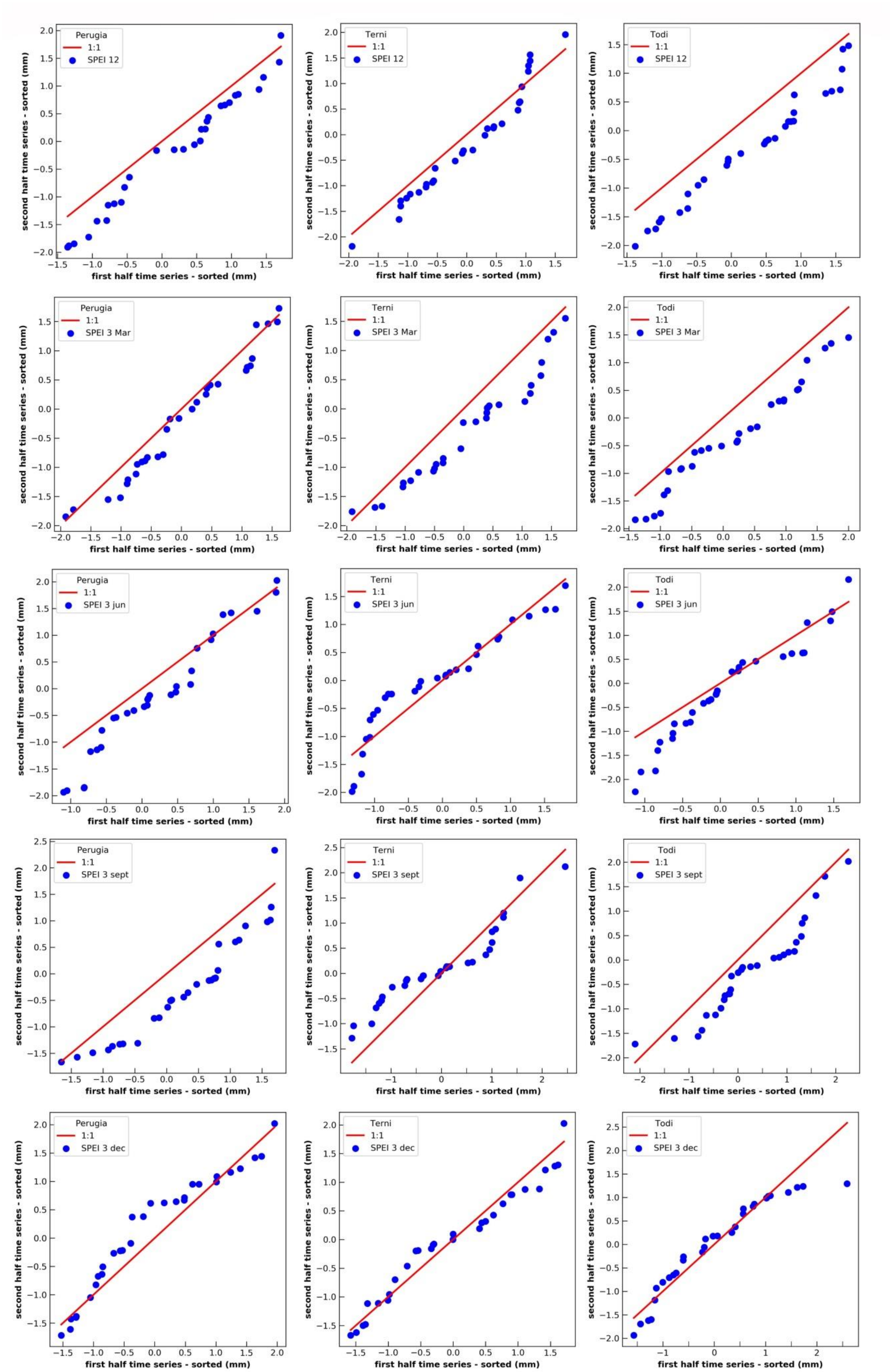

Figure S9. IST method – SPEI 12 and seasonal SPEI 3

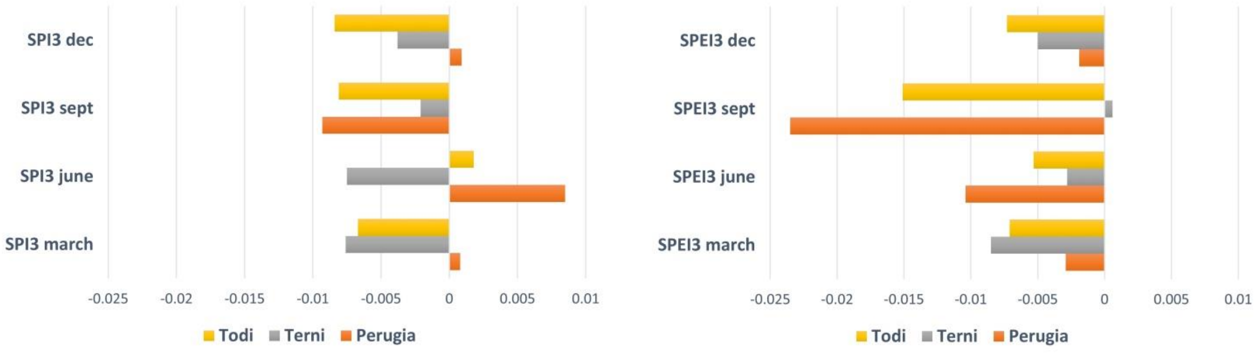

Figure S10. Representation of linear regression slope m in Table 3 of the main article.

S.1.7 Run Test

Table S3: Characterization of drought events for Perugia, Terni, and Todi – time series: SPI12, SPEI12.

Parameters	Perugia				Terni				Todi			
	**spi12**		**spei12**		**spi12**		**spei12**		**spi12**		**spei12**	
	**FP**	**SP**	**FP**	**SP**	**FP**	**SP**	**FP**	**SP**	**FP**	**SP**	**FP**	**SP**
Droughts number	6	8	7	8	6	9	6	8	6	9	6	9
Max droughts duration (years)	5	3	4	4	4	4	4	4	3	8	3	8
Average droughts duration (years)	2.17	1.38	1.71	1.88	2.33	2.00	2.50	2.13	1.50	2.11	1.67	2.33
SD droughts duration (years)	1.60	0.74	1.11	1.13	1.21	1.22	1.22	1.25	0.84	2.26	0.82	2.24
Max Peak Value	1.83		1.91		2.37		2.19		2.64		2.02	
Max Peak Value - occurrence	1985		2017		2007		2007		2011		2011	
Max Severity Value	4.52		3.70		4.51		3.56		6.12		5.99	
Max Severity Value - occurrence	1970-1974		1987-1990		2000-2003		2000-2003		1988-1995		1988-1995	

Table S4: Characterization of drought events - Perugia – time series: SPI3, SPEI3.

**Perugia**	**spi3_march**		**spei3_march**		**spi3_june**		**spei3_june**		**spi3_sept**		**spei3_sept**		**spi3_dec**		**spei3_dec**	
Parameters	**FP**	**SP**	**FP**	**SP**	**FP**	**SP**	**FP**	**SP**	**FP**	**SP**	**FP**	**SP**	**FP**	**SP**	**FP**	**SP**
Droughts number	4	8	5	6	9	6	11	9	5	8	5	6	5	6	5	6
Max droughts duration (years)	5	7	7	5	6	4	2	4	6	3	4	7	8	4	8	5
Average droughts duration (years)	3.00	2.25	4.00	2.17	1.78	2.00	1.09	2.11	2.80	1.75	2.00	3.50	3.40	2.17	3.40	2.33
SD droughts duration (years)	1.63	1.98	2.24	1.47	1.64	1.26	0.30	1.05	2.17	0.89	1.22	2.17	2.79	1.17	2.79	1.51
Max Peak Value	2.65		1.91		1.74		1.94		2.34		1.67		1.75		1.72	
Max Peak Value - occurrence	1993		1990		2006		2017		1988		2018		1973		2006	
Max Severity Value	10.91		9.79		3.76		4.80		6.80		6.62		7.58		7.31	
Max Severity Value - occurrence	1988-1994		1988-1994		1962-1967		2005-2007		1985-1990		2007-2013		1967-1974		1967-1974	

Table S5: Characterization of drought events - Terni – time series: SPI3, SPEI3.

**Terni**	**spi3_march**		**spei3_march**		**spi3_june**		**spei3_june**		**spi3_sept**		**spei3_sept**		**spi3_dec**		**spei3_dec**	
Parameters	**FP**	**SP**	**FP**	**SP**	**FP**	**SP**	**FP**	**SP**	**FP**	**SP**	**FP**	**SP**	**FP**	**SP**	**FP**	**SP**
Droughts number	6	5	7	5	11	8	10	6	10	9	10	8	8	6	9	7
Max droughts duration (years)	3	16	11	4	3	3	3	4	2	2	3	5	6	3	6	4
Average droughts duration (years)	1.67	4.40	3.14	2.00	1.55	2.00	1.60	2.50	1.30	1.44	1.50	2.00	1.88	1.83	1.89	2.14
SD droughts duration (years)	0.82	6.50	3.58	1.22	0.82	1.07	0.84	1.05	0.48	0.53	0.71	1.60	1.73	0.75	1.62	1.07
Max Peak Value	2.37		1.91		2.76		1.99		2.62		1.77		2.07		1.67	
Max Peak Value - occurrence	1993		1990		2006		2006		1988		1988		2007		2011	
Max Severity Value	15.43		11.03		4.21		3.83		3.77		3.07		2.07		7.33	
Max Severity Value - occurrence	1988-2003		1988-1998		2005-2007		2005-2007		1987-1988		1987-1988		2006-2007		1969-1974	

Table S6: Characterization of drought events - Todi – time series: SPI3, SPEI3.

**Todi**	**spi3_march**		**spei3_march**		**spi3_june**		**spei3_june**		**spi3_sept**		**spei3_sept**		**spi3_dec**		**spei3_dec**	
Parameters	**FP**	**SP**	**FP**	**SP**	**FP**	**SP**	**FP**	**SP**	**FP**	**SP**	**FP**	**SP**	**FP**	**SP**	**FP**	**SP**
Droughts number	6	9	6	9	9	8	10	9	9	5	9	8	4	6	6	6
Max droughts duration (years)	3	11	3	11	7	4	7	4	4	7	2	5	6	5	6	6
Average droughts duration (years)	1.83	2.44	1.83	2.44	1.89	1.75	1.70	1.89	1.67	3.80	1.33	2.38	3.50	2.17	2.67	2.33
SD droughts duration (years)	0.98	3.24	0.98	3.24	1.96	1.16	1.89	1.17	1.00	2.39	0.50	1.30	1.91	1.47	1.97	1.86
Max Peak Value	2.40		1.84		1.85		2.26		2.07		2.10		2.52		1.93	
Max Peak Value - occurrence	1993		2012		2006		2003		1985		1985		2011		2011	
Max Severity Value	10.43		10.76		2.72		3.36		4.00		3.89		6.39		6.51	
Max Severity Value - occurrence	1988-1998		1988-1998		1993-1995		2005-2007		2007-2013		1990-1994		1969-1974		1969-1974	

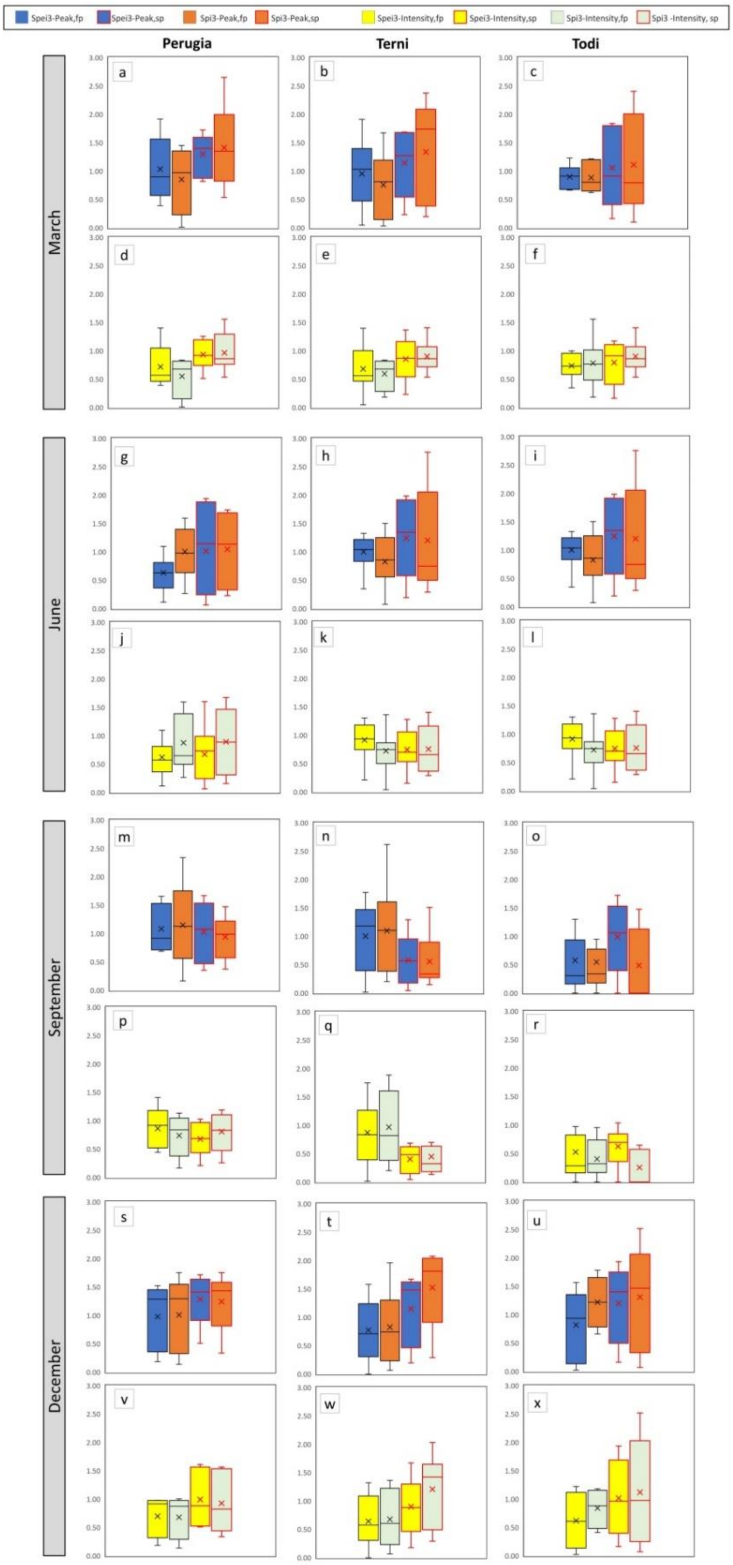

Figure S11: For each station, a boxplot of the absolute value of peak and intensity for SPEI(SPI) 3 (March, June, September, December). Boxplot elements: box = values of 25th and 75th percentiles; horizontal line = median; cross = average; whiskers limit = minimum/maximum value; interquartile range (IQR)= difference between 25th and 75th percentile.

S.1.8 SPEI and SPI correlation

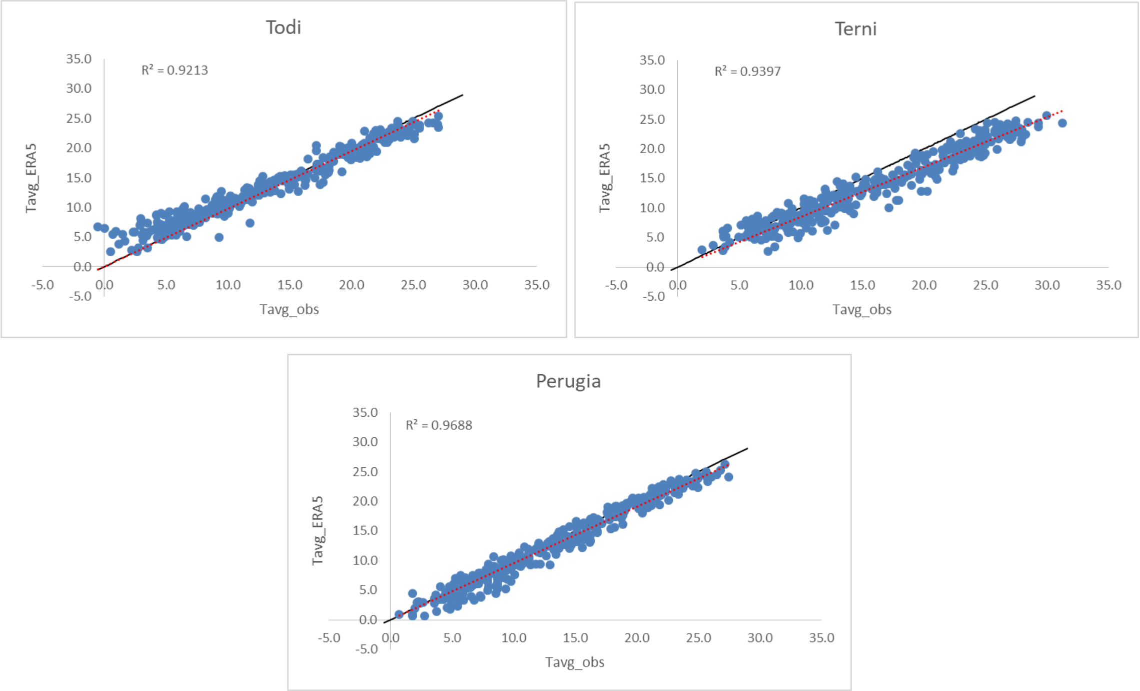

Figure S12. Linear regression – Observed time series of daily average temperature vs. ERA 5 reanalysis extracted data (example plotted for year 1975 for the four meteorological stations). The continuous line and dot line represent the 1:1 line and linear regression line, respectively.

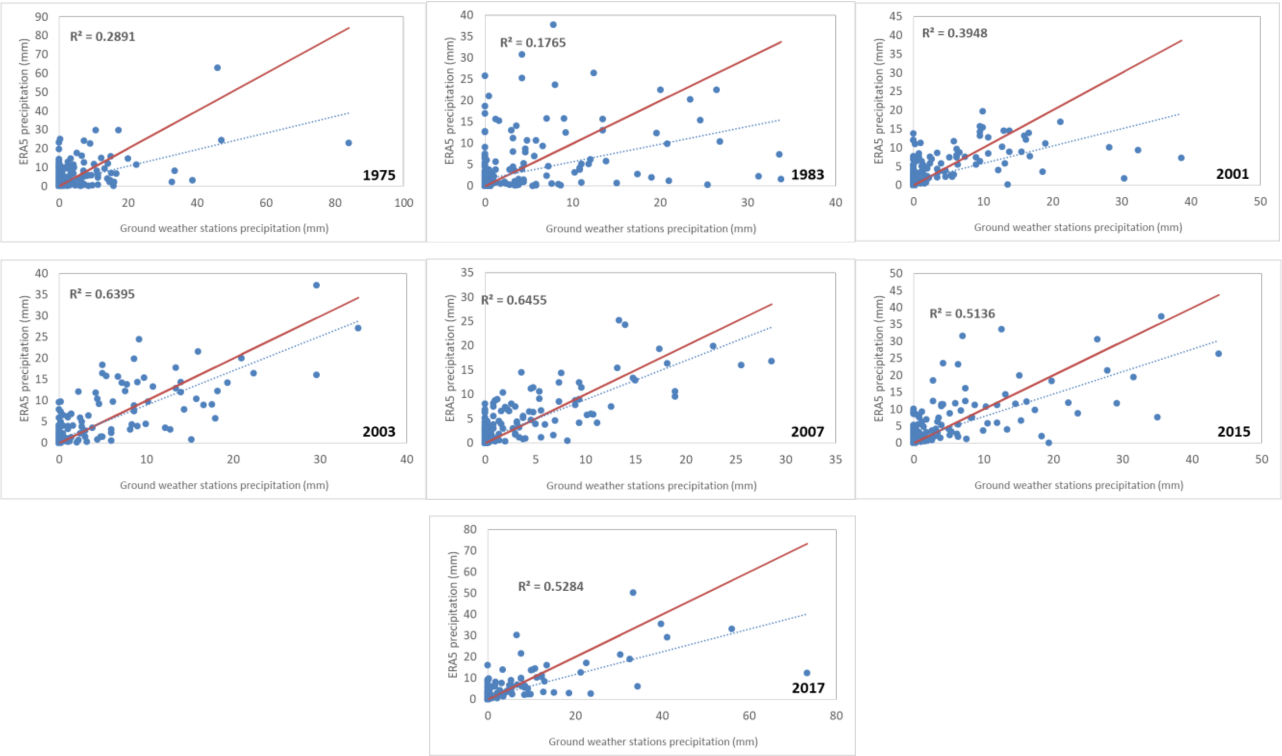

Figure S13. Linear regression – Observed time series of daily average precipitations vs. ERA 5 reanalysis extracted data (example plotted for Terni station for the years with severe droughts from 1961 to 2020). The continuous line and dot line represent the 1:1 line and linear regression line, respectively.

Indicators

The **Pearson correlation coefficient** r was computed using the following equation:

$$r = \frac{\sum_{i=1}^{n}(x_i - \underline{x})(y_i - \underline{y})}{\sqrt{\sum_{i=1}^{n}(x_i - \underline{x})^2}\sqrt{\sum_{i=1}^{n}\left(y_i - \underline{y}\right)^2}} \quad (19)$$

r was used to compare the values of SPI and SPEI, to measure the strength of the linear relationship between the two indices (Tirivarombo et al., 2018). Pearson correlation coefficient r can assume values in a range from -1 to +1: a correlation coefficient of zero indicates that there is no association between variables; the closer the r coefficient approaches +1 (- 1), regardless of the sign, the stronger is the existing association. A r value between 0.68 and 1 is supposed to indicate a strong correlation, but the correlation coefficient is an abstract measure, and a precise and direct interpretation sounds difficult (Taylor, 1990). If the correlation is between only two variables, the values of the Pearson correlation coefficients can be obtained as the root square of the determination coefficient R: $r = \sqrt{R^2}$.

Willmott's index (1981) - *d* is an indicator that is sensitive to variations between measured and modeled values and can reflect the degree to which the model can capture the measured variance (AgriMetSoft, 2019).

$$d = 1 - \frac{\sum_{i=1}^{n}(Obs_i - Sim_i)^2}{\sum_{i=1}^{n}\left(\left|Sim_i - \overline{Obs}\right| + \left|Obs_i - \overline{Obs}\right|\right)^2}, \quad 0 \le d \le 1 \quad (20)$$

References

1. Abramowitz, M., and I. A. Stegun, 1965: Handbook of Mathematical Functions, with Formulas, Graphs, and Mathematical Tables. Dover Publications, 1046 pp.
2. AgriMetSoft (2019). Online Calculators. Available on: https://agrimetsoft.com/calculators/Nash%20Sutcliffe%20model%20Efficiency%20coefficient

3. Beguería S, Vicente-Serrano SM, Reig F, Latorre B. 2014. Standardized precipitation evapotranspiration index (SPEI) revisited: parameter fitting, evapotranspiration models, tools, datasets and drought monitoring. International Journal of Climatology, 34(10): 3001-3023.
4. Dabanlı, İ., Şen, Z., Yeleğen, M. Ö., Şişman, E., Selek, B., & Güçlü, Y. S. Trend assessment by the innovative-Şen method. Water resources management, 30(14), 2016, 5193-5203.
5. Edwards, D.C. and McKee, T.B. (1997) Characteristics of 20th Century Drought in the United States at Multiple Time Scales. Climatology Report 97-2, Department of Atmospheric Science, Colorado State University, Fort Collins.
6. Hargreaves, G.H., Allen, R.G., 2003. History and evaluation of Hargreaves evapotranspiration equation. J. Irrigat. Drain. Eng. 129, 53–63. https://doi.org/ 10.1061/(ASCE)0733-9437(2003)129:1(53).
7. Hosking JRM. 1986. The theory of probability weighted moments. Res. Rep. RC 12210 IBM Research Division, Yorktown Heights NY 10598.
8. Nash, J. E. and Sutcliffe, J. V. (1970) River flow forecasting through conceptual models part I — A discussion of principles, J. Hydrol., 10(3), 282–290, doi:10.1016/0022-1694(70)90255-6,
9. Pieper, P., Düsterhus, A., & Baehr, J. (2020). A universal Standardized Precipitation Index candidate distribution function for observations and simulations. Hydrology and Earth System Sciences, 24(9), 4541-4565.
10. Sen, Z. Innovative trend analysis methodology. Journal of Hydrologic Engineering, 17(9), 2012, 1042-1046.
11. Sen, Z. Innovative trend significance test and applications. Theoretical and applied climatology, 127(3), 2017, 939-947.
12. Singh, V.P., Guo, H., Yu, F.X., (1993). Parameter estimation for 3-parameter log-logistic distribution (LLD3) by Pome. Stochastic Hydrology and Hydraulics, 7, 163-177.
13. Svoboda M., Hayes M., Wood D. Standardized Precipitation Index User Guide. World Meteorological Organization, Geneva, 2012.
14. Taylor R., Interpretation of the Correlation Coefficient: A Basic Review. Journal of Diagnostic Medical Sonography. 1990;6(1):35-39. doi: 10.1177/875647939000600106
15. Thom, H. C. S., 1966: Some Methods of Climatological Analysis. WMO Technical Note Number 81, Secretariat of the World Meteorological Organization, Geneva, Switzerland, 53 pp.
16. Vicente-Serrano, Sergio M.; Beguería, Santiago; López-Moreno, Juan I. A multiscalar drought index sensitive to global warming: the standardized precipitation evapotranspiration index. Journal of climate, 2010, 23.7: 1696-1718.
17. Wu, H., Svoboda, M.D., Hayes, M.J., Wilhite, D.A. and Wen, F. (2007) Appropriate application of the standardized precipitation index in arid locations and dry seasons. International Journal of Climatology, 27(1), 65–79. https://doi.org/10.1002/joc.1371.
18. Yang, Y., Chen, R., Han, C., & Liu, Z. (2021). Evaluation of 18 models for calculating potential evapotranspiration in different climatic zones of China. Agricultural Water Management, 244, 106545.
